# STDP Allows Fast Rate-Modulated Coding with Poisson-Like Spike Trains

**DOI:** 10.1371/journal.pcbi.1002231

**Published:** 2011-10-27

**Authors:** Matthieu Gilson, Timothée Masquelier, Etienne Hugues

**Affiliations:** 1 Department of Electrical and Electronic Engineering, The University of Melbourne, Melbourne, Australia; 2 Lab for Neural Circuit Theory, Riken Brain Science Insitute, Wako-shi, Saitama, Japan; 3 Unit for Brain and Cognition, Universitat Pompeu Fabra, Barcelona, Spain; University of Edinburgh, United Kingdom

## Abstract

Spike timing-dependent plasticity (STDP) has been shown to enable single neurons to detect repeatedly presented spatiotemporal spike patterns. This holds even when such patterns are embedded in equally dense random spiking activity, that is, in the absence of external reference times such as a stimulus onset. Here we demonstrate, both analytically and numerically, that STDP can also learn repeating rate-modulated patterns, which have received more experimental evidence, for example, through post-stimulus time histograms (PSTHs). Each input spike train is generated from a rate function using a stochastic sampling mechanism, chosen to be an inhomogeneous Poisson process here. Learning is feasible provided significant covarying rate modulations occur within the typical timescale of STDP (∼10–20 ms) for sufficiently many inputs (∼100 among 1000 in our simulations), a condition that is met by many experimental PSTHs. Repeated pattern presentations induce spike-time correlations that are captured by STDP. Despite imprecise input spike times and even variable spike counts, a single trained neuron robustly detects the pattern just a few milliseconds after its presentation. Therefore, temporal imprecision and Poisson-like firing variability are not an obstacle to fast temporal coding. STDP provides an appealing mechanism to learn such rate patterns, which, beyond sensory processing, may also be involved in many cognitive tasks.

## Author Summary

In vivo neural responses to stimuli are known to have a lot of variability across trials. If the same number of spikes is emitted from trial to trial, the neuron is said to be reliable. If the timing of such spikes is roughly preserved across trials, the neuron is said to be precise. Here we demonstrate both analytically and numerically that the well-established Hebbian learning rule of spike-timing-dependent plasticity (STDP) can learn response patterns despite relatively low reliability (Poisson-like variability) and low temporal precision (10–20 ms). These features are in line with many experimental observations, in which a poststimulus time histogram (PSTH) is evaluated over multiple trials. In our model, however, information is extracted from the *relative* spike times between afferents without the need of an *absolute* reference time, such as a stimulus onset. Relevantly, recent experiments show that relative timing is often more informative than the absolute timing. Furthermore, the scope of application for our study is not restricted to sensory systems. Taken together, our results suggest a fine temporal resolution for the neural code, and that STDP is an appropriate candidate for encoding and decoding such activity.

## Introduction

STDP is now a well-established physiological mechanism of activity-driven synaptic regulation [1], which can capture spiking information at a short timescale, down to milliseconds [2], [3]. Although the relationship between the stimulating input structure and the resulting weight specialization has been investigated in a number of theoretical studies [4], [5], [6], [7], most of them have limited their scope to general and abstract input structures.

A practical and fundamental question is to understand how, in natural or experimental situations, STDP can participate in the learning process. Importantly, although repeated stimulus presentations, or task trials, induce memorization (e.g. [8]), the underlying neural mechanisms remain largely unknown. In this respect, a recent numerical study showed that a repeating arbitrary, but reliable, spatiotemporal spike pattern embedded in equally dense random activity can be learned and robustly detected by a single neuron equipped with STDP [9]. However, such reliable spike patterns have received scarce experimental evidence (but see [10], [11], [12], [13], [14], [15], [16]) and may constitute a very special case of activity. More generally, and across trials, spike trains often exhibit large variability, and can be described using an underlying probabilistic firing intensity, for example through inhomogeneous Poisson sampling. This hypothesis is tenable with most – if not all – experimental datasets, where the temporal spiking probability – or rate – is measured by a post stimulus time histogram (PSTH) [17]. PSTHs usually exhibit temporal peaks, whose spread width is of the order of ∼10–20 ms in many experimental findings [18], [19], [20], [21], [22], [23], [24], [25], [26]. Whether STDP is able to learn such rate patterns is currently unclear. Somewhat surprisingly, our study shows that such spread widths and Poisson-like firing variability are not an obstacle to STDP-based pattern learning, and fast and efficient detection afterwards.

**Figure 1:**
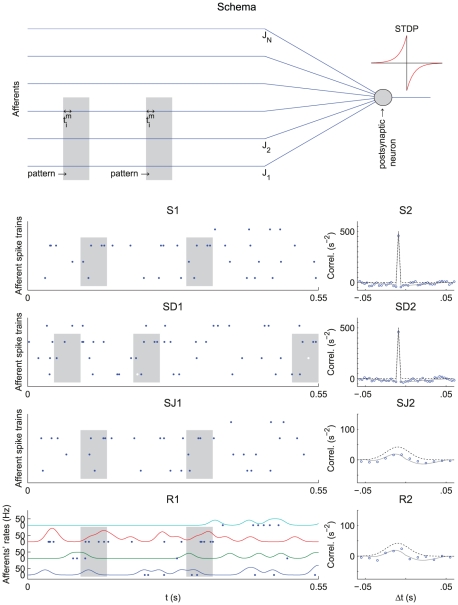
Pattern models and associated cross-correlograms. Schema: representation of 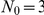 pattern (bottom) and 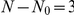 non-pattern (top) inputs that excite, through synapses with weights 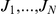, a postsynaptic neuron equipped with STDP. Grey areas indicate the pattern presentations. For afferent 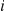, 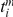 denotes the latency of the 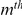 spike (Model S) or rate peak (Model R). Below, the left panels (label 1) show the raster plots (each dot indicates a spike) for 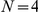 afferents, involving 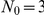 pattern inputs. The right panels (label 2) compare predictions (dotted and dashed lines) that correspond to Equation (1) and numerical simulations (circles) for the correlograms. The dashed lines involve an additional approximation compared to the dotted line that is more accurate (compare Equations (S11) in Text S1 and (13), respectively). All patterns have the same latencies 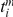. (S) Model S with 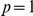, no jitter and 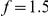 Hz. Spike times (dots) are the same across presentations for the three pattern inputs (bottom), but not for the fourth non-pattern input (top). The cross-correlogram between second and first afferent exhibits a peak of height 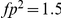 Hz at 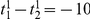 ms, cf. Equation (13). (SD) Model S with 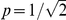 (notice the missing spikes, and the ones added to compensate, in white) and 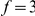 Hz, other parameters being the same as in (S). The correlogram is similar to that in (S), in particular its height equal to 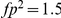 Hz. (SJ) Model SJ with Gaussian jittering at each presentation with spread width 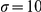 ms. The correlogram has a peak centered at 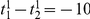 ms and spread width 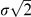, cf. Equation (14). (R) Model R. The rate functions were obtained by convolving the spike trains of (S1) by a Gaussian with amplitude 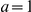 and width 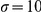 ms (both inside and outside pattern presentations). The rate profiles are thus the same across all pattern presentations for the three pattern inputs (bottom), except for border effects, but not for the non-pattern input (top). From the rate functions, the spikes (dots) are generated using inhomogeneous Poisson processes and thus differ between presentations, both in timing and count. The cross-correlogram between second and first afferents for model R is similar to that in (SJ), cf. Equation (17). The simulated correlograms are averaged over 1000 s for spike patterns (S, SD, SJ) and 50000 s for rate-modulated patterns (R).

We consider a single postsynaptic neuron excited by presynaptic neurons (Fig. 1Schema), of which an arbitrary and hidden number are involved in the repeating presentation of a given pattern, embedded in otherwise random spike trains. A main and novel contribution of this study concerns patterns generated using covariations of the input instantaneous rates, from which spikes are generated through an inhomogeneous Poisson process. To predict the evolution of the synaptic weights and the resulting neuronal selectivity, we analyze theoretically a dynamical system that describes the effect of STDP. We confirm these results using numerical simulations. We demonstrate that repeated presentations of such rate patterns induce spike-time correlations that are captured by STDP, even when rate peaks have a width up to 10–20 ms. In general, STDP favors synapses corresponding to early spikes in the pattern, resulting in fast response whenever the pattern is presented [9]. However, when the pattern exhibits sharp and/or large-amplitude peaks for several inputs, STDP tends to favor some of the corresponding synapses. Besides rate-modulated patterns, our theory also applies to spike patterns that we therefore include here for the sake of completeness.

## Materials and Methods

We first describe the models of patterned activity used to train the neuron. Then, we present the models of STDP and Poisson neuron, which are the basis of the mathematical framework that describes the weight evolution. We build on our previous theoretical work where the synaptic dynamics is governed by the firing rates and spike-time correlations of input spike trains [27]. That framework is adapted to the present situation where spike trains convey repeating patterns, which allows us to predict the neuronal specialization in terms of the pattern, STDP and neuronal parameters.

### From spike patterns to rate-modulated patterns

Recent work has focused on generating spike trains with a given correlation structure, using a mixture of spike coordination and rate covariation [28]. Here these two mechanisms are used to generate each a class of patterns: spike patterns (model S) and rate-modulated patterns (model R), the last mimicking PSTH-like probabilistic spiking activity. Throughout the present paper, we consider a single pattern that is presented to an unknown subset of 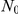 among 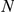 excitatory afferent (or input) plastic synapses that stimulate a single neuron (Fig. 1Schema). The afferents involved and those not involved in the pattern will be denoted by pattern and non-pattern afferents, respectively. Pattern presentations occur randomly with frequency 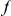 and duration 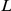, without overlapping. All pattern models rely on latencies 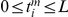 for each pattern afferent 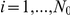 and 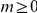. Unless said otherwise (cf. bimodal patterns below), the latencies 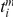 are uniformly distributed in 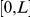, i.e., corresponding to a (single) realization of a homogeneous Poisson process with intensity rate 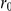 for the duration 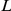 for each input. Once determined these latencies (possibly none) for all pattern inputs, we generate the input spikes for each pattern presentation as follows:

- Model S: **S**pike pattern with fixed latencies. Every latency 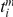 induces a spike with probability 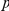 at each pattern presentation; the precise spike time corresponds to the laps 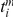 after the start of the presentation. In order to preserve the mean firing rate, spikes generated using a homogeneous Poisson spike train with rate 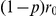 are added. Fig. 1S1 shows an example with 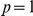 and Fig. 1SD1 an example with 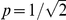 (‘D’, standing for **D**eletion, is used whenever 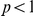).
- Model SJ: **S**pike pattern with **J**ittered latencies (Fig. 1SJ1). This model is derived from model S, such that the spike times are chosen around each latency 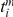 using a Gaussian-distributed jitter with spread width 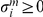. Simulations will often use a common value 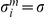. We will only use 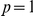 in this case. Fig. 1SJ1 shows an example with 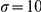 ms.
- Model R: Poisson **R**ate-modulated pattern (Fig. 1R1). Inhomogeneous Poisson sampling is used to generate the spike times at each presentation. For pattern afferent 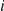, the corresponding instantaneous rate intensity is generated using the latencies 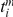, each being the center of a Gaussian kernel function with spread width 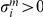 and total area 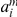 (the Gaussian peak alone has a maximal height 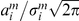). We will often use a normalized amplitude (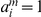), such that the resulting mean firing rate is comparable to patterns of models S with the same latencies, as well as a common spread width 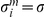 for all spike trains.

For each of the 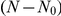 non-pattern inputs, as well as the 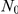 pattern inputs *outside* pattern presentations, latencies similar to 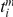 are generated using a homogeneous Poisson process with rate 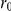; then the same spike generation applies according to each model. Note that the choice of Gaussian functions in model R is motivated by analytical tractability, but any peaked function could be used. We did not consider a probability of occurrence for the Gaussian peaks in model R (similar to 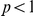 in model SD) as variability was already present in the spike generation.

In the remainder of the present paper, we sometimes refer to models S in general as spike patterns, which include model SJ, in contrast to model R.

In the baseline simulations, and unless stated otherwise, we use 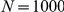, 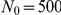, 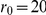 Hz, 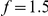 Hz and 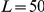 ms.

### Phenomenological model of STDP

We use an abstract model of STDP where the weight change depends on the relative spike timing and the current value for the weight. In our model, each single spike and each pair of presynaptic and postsynaptic spikes contribute once to plasticity. A sole pair with respective times 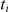 and 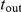 induces a weight change 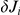 determined by the following contributions
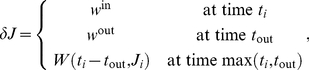
(1)The rate-based contributions 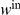 and 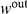 account for the (one-shot) effect of each pre-and postsynaptic spikes, respectively [4], [27]. They model homeostatic synaptic scaling mechanisms [29] (see also Discussion) and allow us to examine theoretically the weight specialization depending on individual spikes, while evacuating rate effects [27]. Those terms are not seen in typical STDP experiments, but they could be easily missed if its magnitude is lower than the STDP changes. Theoretically, 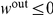 can be chosen equal to zero provided depression dominates the STDP effects. Even though there exists a parameter range for which rate effects are small (or even balance each other) and STDP dominates the plasticity effects, we have used values for these rate-based coefficients that gave very robust results without any fine-tuning in our baseline numerical simulations.

**Figure 2:**
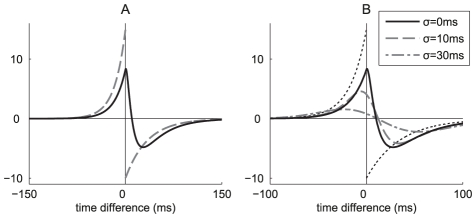
Effective STDP learning window. (A) Plot of the functions 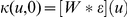 in Equation *(30)* (solid line) and the STDP learning window function 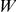 (dashed line). (B) Plots of the function 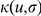 for 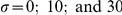 ms.

The STDP learning window function 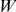 describes the effect of long-term potentiation (LTP) and long-term depression (LTD) on the weight 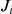 as a function of the spike-time difference 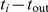 and weight 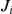. The learning rate 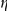 determines the speed of learning. We consider a classic learning window function determined by a decaying exponential 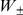 for each side (see dashed line Fig. 2A):
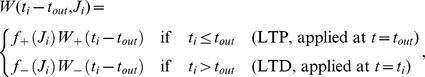
(2)where 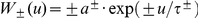 fits the mean effect observed in experimental data [30]; a complete list of parameters can be found in Text S1 (Section S3.2). The scaling functions 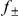 determine the dependence of the change in the weight on its current value [31]. In the analysis and most numerical simulations, we use additive STDP, for which these functions are constant, namely 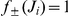, like in [9], which leads to bimodal weight distributions [32], and therefore strong resulting selectivity. However a slightly multiplicative STDP can be of interest, because it can ensure both competition and homeostasis [33], without the need for the additional rate-based homeostatic terms (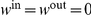). This will be used in the Results section (‘Influence of the STDP and neuronal parameters’) with the model proposed in [6] for which a parameter 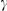 scales between additive (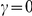) and multiplicative (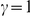) STDP:
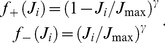
(3)The constant 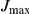 is the upper “soft” bound, while the lower bound is set to zero.

However, a too strong weight dependence weakens (and eventually impairs) the resulting specialization [6], [33].

### Poisson neuron model

The Poisson neuron [4] is an abstract neuronal model where the spiking mechanism that generates the spike train 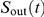 is approximated by an inhomogeneous Poisson process. The latter is driven by a (positive) rate function that mimics the mean potential of a soma receiving pre-synaptic activity:
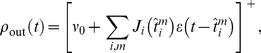
(4)where 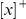 is the positive part: 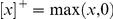. The time course of each EPSP following the 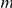-th spike arriving at synapse 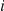 at time 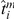 is described by the kernel function 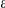 rescaled by the weight 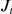. The constant 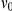 relates to other non-plastic input connections that are not considered in detail; they can be excitatory or inhibitory. Our analysis assumes 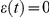 for 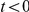 in order to preserve causality and 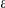 to be normalized: 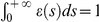. Note that the calculations are exact only when the soma potential is positive at all times. Numerical simulations use 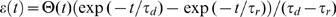, where 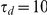 ms and 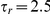 ms are the decaying and rising time constants, respectively; 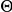 denotes the Heaviside step function.

To minimize false alarms in a detection scheme using a single neuron, we have used inhibition (background activity 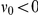), which leads to a subthreshold regime where the postsynaptic neuron has a low output firing rate. This complies with *in vivo* experiments where neurons receive excitatory and inhibitory inputs that almost balance each other [34] or even favor inhibition [35]. Using strong inhibition, a single Poisson neuron can be trained to be almost as reliable as a deterministic LIF neuron for pattern detection (see Results section).

### Evolution of the synaptic weights

Our choice for the models of additive STDP and Poisson neuron allows us to derive a dynamical system to analytically examine the weight dynamics. We draw on a previously developed framework [4], [27], where details can be found. Under the assumption of slow learning compared to other (firing and synaptic) mechanisms, an intermediate averaging period 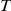 can be chosen between the two corresponding time scales. The expectation of the weight update corresponding to Equation (1) over the period 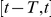 can be evaluated using the firing rates and spike-time covariance of the input and output spike trains:
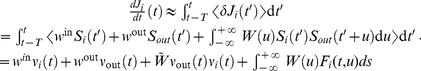
(5)


Here spikes are considered to be quasi-instantaneous events, so the spike trains 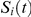 and 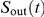 for of input 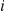 and the neuron are modeled as a sum of delta functions (Dirac combs). The angular brackets denote the ensemble average over the randomness of the spike trains, since we generate external inputs using stochastic processes. In Equation (5), the terms 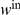 and 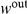 are associated with the mean (time-averaged) firing rates of the 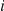-th afferent and neuron, 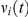 and 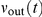, respectively, which are defined similar to:
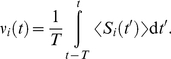
(6)


The contributions of spike pairs that involve the STDP learning window function 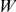 in Equation (1) is decomposed into two terms (on the last line). The first one gives the product of the pre- and postsynaptic firing rates with 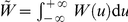, the integral value of 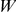. The second one gives the convolution of 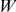 with the neuron-to-input (time-averaged) cross-covariance between the neuron and input 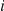:
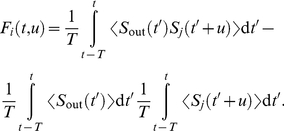
(7)


The Poisson neuron described by Equation (4) leads to the following consistency equations for the neuronal output firing rate and neuron-to-input covariance, respectively:
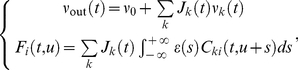
(8)where the input-to-input (time-averaged) cross-covariance is defined by
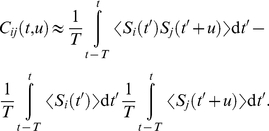
(9)


Equations (8) are exact only when the soma potential in Equation (4) is positive at all times; this is of importance when considering negative values for the constant 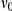. As will be justified in the following section, the input firing rates in Equation (6) and spike-time covariance in Equation (9) are independent of time 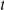, so we omit the latter variable thereafter (except for the plastic weights when it is useful to precise). We combine Equations (5) and (8) to obtain Equation (29) in Results. Using matrix notation, it can be rewritten as a linear differential for the drift (first stochastic moment) of the synaptic weights in terms of the input properties:
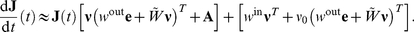
(10)


The 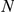-column vector 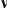 contains the input firing rates 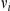; 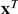 denotes the transpose of 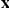; 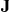 is the 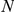-row vector of the weights; we have also defined 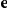 as the 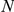-column vector that has all its elements equal to one. The matrix 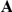 absorbs the input correlations, neuronal and STDP parameters
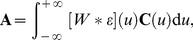
(11)where 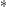 denotes the usual convolution of functions.

### Repeating patterns and input spike-train structure

The previous section showed how the weight evolution defined in Equation (1) is determined by the input firing rates in Equation (6) and spike-time covariances embodied in the coefficients of matrix 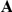 in Equation (11). In order to predict the weight evolution, we need to evaluate the respective variables 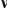 and 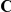 for input spike trains that convey pattern activity. The present study compares the two classes of patterns described at the beginning of Materials and Methods: model S with coordination of spike times; and model R with covariation of firing rates for inhomogeneous Poisson processes. We provide details of the calculations that lead to the following results in Text S1 (Section 1).

In all the pattern models considered throughout this paper, input spike trains have a quasi-constant mean firing rate as defined in Equation (6). This follows because we consider relatively short patterns (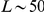 ms) compared to the averaging period 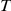 and that the pattern spikes (per input) correspond to a firing rate comparable to the background rate. We focus on the “difficult” situation where the frequency of the pattern presentations is not too high, such that the condition 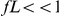 is satisfied. In this case, the discrepancies between the numbers of pattern spikes for different inputs and different presentations do not affect the mean firing rates, which are almost identical for all inputs (and roughly equal to the background rate):
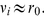
(12)


For rate-modulated patterns of model R, Equation (12) requires that all rate peaks are normalized (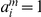). Detailed calculations are provided in Text S1 (Section 1.1).

Similar to the mean firing rates 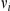, the mean covariances 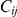 are also practically independent of time 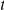. In spike patterns of model S (with no jitter), pattern inputs repeatedly and consistently fire spikes with given latencies. Consequently, each pair of pattern input spike trains involves synchrony with time lags that are determined by the relative spike latencies. In other words, the corresponding spike-time correlogram 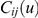 defined in Equation (9) exhibits a peak for those preferred time lags, as detailed in Text S1 (Section 1.2). Namely, for two pattern inputs 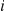 and 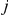 with respective latencies 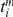 and 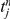, we have
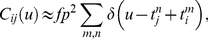
(13)where 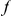 is the frequency of the pattern presentation and 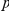 is the probability for a spike at each latency to be fired during a pattern presentation. The approximation in Equation (13) neglects a term related to the “silence” for pattern inputs during the pattern presentation beside the spikes at latencies 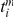. The same term applies to all pairs among the 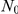 pattern inputs and can thus be ignored when studying the emerging weight structure; this also partly explains discrepancies between theoretical predictions and numerical simulations (see “Weight specialization by competition”). Jittering the spike times around the mean latencies 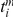 amounts to replace the delta function in Equation (13) by the convolution of the jitter distributions. Consequently, Gaussian jitters with spread widths 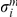 in model SJ lead to:
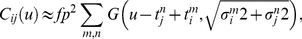
(14)where 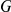 is the normalized Gaussian function of width 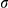:
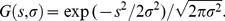
(15)


Details are provided in Text S1 (Section 1.3).

Model SB is a particular case of model S, where inputs are partitioned into two groups. Each input 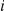 in group 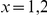 has a single latency 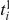 generated by a Gaussian distribution that is common to all inputs from the same group, namely with mean latency 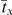 and variance 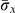. Recall that we constrain this special case of model S to 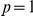 and no jitter (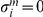). After population average (denoted by the overline), the mean cross-correlogram between input groups 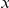 and 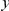 in Equation (13) becomes:
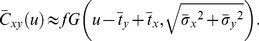
(16)


Details are provided in later in Text S1 (Section 1.5). In a sense, the randomness over each population plays the same role as individual jitters in Equation (14).

For patterns of model R, the covariances are given in a similar manner by the convolution of the Gaussian kernels that determine the rate covariations for each spike train (Fig. 1R1):
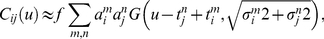
(17)where 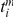, 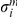 and 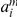 are the center, the width and the amplitude, respectively, of the corresponding rate peaks. Note that we take into account the whole Gaussian functions even outside the pattern of duration 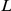. See Text S1 (Section 1.4) for details.

The expressions in Equations (13), (14) and (17) are actually particular cases of the same general formulation given in Equation (28) in Results. Once known the input firing rates and spike-time correlations for a given pattern, the weight dynamics can be analyzed using Equation (10).

### Homeostatic equilibrium

We require STDP to produce a stable equilibrium for the mean input weight 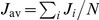, which also stabilizes the neuronal output firing rate. This favors an effective weight specialization when maintaining the mean weight between the lower and upper bounds, which allows to potentiate (select) some weights while depressing (discarding) others. As detailed in Text S1 (Section 2.1), we average Equation (10) and ignore matrix 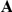 to evaluate the dynamics of the mean weight. Note that this is equivalent to averaging over all inputs Equation (29) in Results and neglecting the correlation terms involving 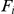. The conditions
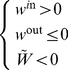
(18)ensure stable fixed points both for 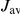 at the equilibrium value
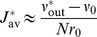
(19)and output neuronal firing rate at
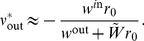
(20)


For the equilibrium to be realizable, the equilibrium value 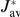 must be within the weight bounds (e.g., Fig. S1 in Text S1), which implies in particular that 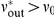; a sufficiently large value for 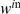 can ensure this condition to be satisfied [4], [27] or a negative value 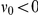 can be used, as chosen here. Here the equilibrium is homeostatic [29] in the sense that the constraint in Equation (19) scales up the weights if their number decreases in order to maintain the level of the neuronal firing rate. This also guaranties that the neuron will not become silent when it is stimulated.

### Weight specialization by competition

For sufficiently strong input correlations (i.e., peaked correlograms in the right panels of Fig. 1), the spike-based correlation terms 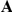 become the leading order in Equation (10), because the other terms roughly cancel each other so long as the homeostatic equilibrium remains satisfied. This causes competition between individual weights, but does not impair the stability of homeostasis.

When the homeostatic equilibrium is realizable, the mean weight 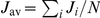 quickly converges close to the predicted equilibrium value 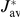 and remains in the vicinity during the whole simulation time. Here the discrepancy of about 15–20% can be explained by the fact that the prediction does not involve the mean input spike-time correlation; cf. Section 2.1 in Text S1 for details. Meanwhile, a portion of individual synapses become potentiated, whereas most of the remainder become depressed almost to zero. The result is a bimodal weight distribution, where selected inputs have potentiated synapses, while the remainder hardly affects the output neuronal firing. A typical example can be found in Fig. S1 in Text S1.

Now we show how to predict such a competition between synaptic weights using Equation (10). We adapt previous results [4], [27] to the present context. The key to predict the weight evolution is thus the spectral analysis of matrix 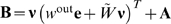. More precisely, the weight specialization is determined by a divergent behavior of individual weights related to the eigenvalues of 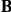 that have a positive real part. Meanwhile, the preservation of the homeostatic equilibrium is ensured by a largely negative (real) eigenvalue of 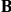 that dominates the spectrum. In the simulations of section “General case of an arbitrary pattern”, this eigenvalue is roughly 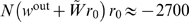, about 100 times larger in magnitude than the other eigenvalues. Due to the fast time scale related to this large negative eigenvalue, we consider that homeostasis is attained well before specialization begins. To ensure a strong neuronal selectivity after learning, we have chosen the parameters such that the equilibrium mean input weight 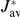 in Equation (19) is low compared to 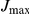. Because this equilibrium value is low compared to the initial distribution (uniform between zero and a maximal value, smaller than the upper bound), almost all weights are depressed at the beginning of the learning epoch. Consequently, we assume the weights to be roughly homogeneous before being specialized. This explains why the initial weight distribution has no impact in the present study. Note that homeostatic mechanisms (in particular 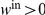 here) save silent synapses from becoming static.

The emerging weight structure is determined by the eigenvalues with largest positive real parts of matrix 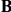, which are actually closely related to those of 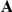 in Equation (11). Therefore, the weight specialization can be predicted with a satisfactory approximation by studying the spectra of 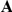 alone. For this purpose, we use the expression in Equation (11) obtained when using the Poisson neuron. For both models S and R, the common expression for their input spike-time correlograms leads to similar dynamics; cf. Equations (28) and (30) in Results. Following Equation (11), the elements of 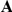 involve the convolution of 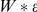 with the Gaussian kernel function 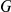 in Equation (15) that describes the temporal variability in our pattern models. This leads to the expression for 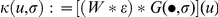 in Equation (31) in Results. It follows that the temporal resolution of the pattern, measured by 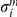, affects the spectrum of 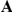, hence the weight dynamics. This effect is verified using numerical simulation in Results. The following section provides a simplified analysis for the weight dynamics for some particular distributions of latencies. However, in the general case, a more complete study of the spectrum of 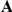 is necessary.

Following this initial splitting, some weights start to win the competition to drive the neuronal output firing. More precisely, inputs with potentiated weights also have stronger cross-covariance with the output spike train in the “causal” range, namely for 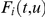 for 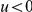. From Equation (8), it is clear that the synaptic weights linearly scale the input cross-covariance structure 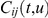 in the expression of the neuron-to-input covariance structure 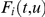. Because of the PSP response (here embodied in the kernel function 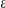), peaks in 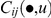 become peaks in 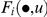, but shifted toward more negative values of 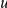, i.e., toward the causal side. Actually, 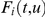 is related to the driving of the neuronal output firing by input 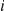 via peaks (or positive values) for 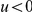. In other words, these correspond to spikes that perdict the neuronal output firing in the next instant [3] It follows that an STDP rule that induces potentiation for causal firing (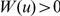 for 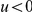) results in more potentiation for the weights that are already strong (and thus take a good part in driving the neuron).

In the case of additive STDP, groups of weights diverge apart from each other until saturation at the upper bound or fading to zero. Then, the choice of the weight bounds affects the learned selectivity of the neuron. Since almost all weights asymptotically become either quiescent at zero or saturated at 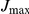, the number of potentiated weights is roughly 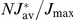 (provided the homeostatic equilibrium is realizable). The portion of selected pattern inputs can thus be adjusted via the equilibrium value in Equation (19). The previous study by Masquelier and colleagues used 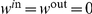 [9], for which the equilibrium is not realizable. It follows that all input weights tend to be depressed so long as the neuron is not silent. Stronger input correlations (e.g., more frequent pattern presentations) and tuning the STDP parameters are then necessary to obtain effective learning; further details are discussed in Text S1 (Section S2.4). For weight-dependent STDP, the above-mentioned trends are still qualitatively valid, even though the fixed point for the mean weight 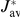 is also determined by the scaling functions 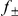 in Equation (1) and individual weights saturate at stable intermediate values between the bounds. A too strong weight dependence of the learning window may prevent the splitting of the weight distribution [6] and thus compromise the resulting neuronal specialization.

### Potentiation of inputs corresponding to early pattern spikes

Until now, the analysis did not assume any specific distribution of latencies for the pattern models (either S or R), except that the number of pattern spikes for each pattern input roughly corresponds the time-average input firing rate. Now we consider the default case of uniformly distributed latencies during the pattern presentation. We show that a general trend arise because of the temporal (approximate) antisymmetry of the STDP learning window considered here, namely inputs with early spikes are more likely to be selected compared to others.

When 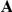 has certain properties, the weight evolution can be described in a more intuitive way than studying its full spectrum. For this alternative analysis, we assume identical input firing rates and homogeneous initial weights. Following a previous study [4], the relative evolution of two input weights 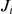 and 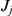 can be evaluated using the reformulation of the learning equation (29) in Results. Namely, the difference between their derivatives yields
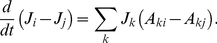
(21)


If, for example, each matrix element of the column for input 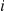 is stronger than the corresponding element for input 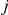, namely
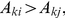
(22)then 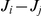 will increase. Because the homeostatic equilibrium remains satisfied during the weight specialization (due to the large negative eigenvalue of 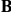 that dominates the remainder of the spectrum), the sum of all input weights is constrained to 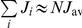. Combining these two trends, when inputs can be divided into two groups of inputs (say, with respective indices 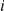 and 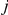) such that Equation (22) holds for all indices in each group, the weights 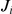 are potentiated whereas 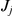 are depressed.

Now we consider patterns of either model S or R for which the latency for each input is chosen randomly with uniform density over the pattern duration. In other words, all latencies can be found across the population of inputs. In this case, the effect of STDP arises from the temporal antisymmetry of 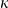 in Equation (31) and Fig. 2, itself related to our choice for 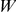. For a relatively short pattern duration 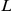, each pattern input 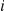 has only a few spikes or peaks with latencies 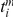. In the simple case of a single pattern spike per input, the contributions to the elements of 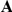 are more likely to be positive for early latencies 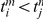, since we have then for most 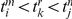:
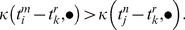
(23)


As a general trend that extends the explanation above, when input 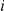 has earlier spikes than input 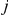, Equation (23) is satisfied for most indices 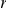 and input 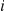 should win the competition over input 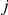. Further developments of this argument are presented in Text S1 (Section S2.2) for the specific patterns of models SB and RB that are examined in the section “Influence of the spike distribution within the pattern”.

A subsequent effect has been demonstrated in a previous numerical study [9]: when some weights saturate (or are significantly large), inputs with spikes that come earlier tend to be potentiated. A Poisson neuron is more likely to fire a postsynaptic spike after each input spike cluster that corresponds to potentiated weights, which explains this reduction of the firing latency. For the deterministic LIF neuron, this effect is more pronounced. However, this prediction may not be valid for a general pattern, in particular with an inhomogeneous spike distribution (see Sections ‘Influence of the spike distribution within the pattern’ and ‘General case of an arbitrary pattern’ in Results); therefore, we did not develop the theory in that direction.

A previous study used similar techniques to investigate the weight dynamics for a general class of time-varying inputs [36]. Apart from the specific input spike trains considered here, a crucial difference here lies in our use of the temporal (approximate) antisymmetry of function 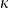 to extract the spiking information (correlations) from the pattern. In the present study, the weight dynamics is very robust, since 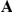 then contains both positive and negative matrix elements and Equation (10) has a stronger drift. If function 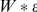 is rather symmetrical as considered by [36], early spikes are not predicted to be potentiated, but patterns may still be learnable.

### Fano factor of the spike count for spike trains of model SD

For a given pattern input 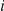 of model SD, we denote by 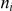 the number of spikes involved in the pattern presentation. Each spike has probability 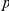 to occur, leading to a number of spikes 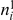 that follows a binomial distribution, with mean 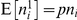 and variance 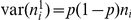. To compensate the missing spikes, a homogeneous Poisson spike train with rate 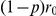 is added to the aforementioned spike train. Because the pattern presentation has duration 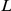, the number 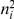 of additional spikes has the same mean and variance 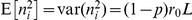. The random variables 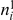 and 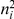 being independent, the total spike count 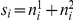 during pattern presentation gives the following Fano factor for the spike train of input 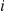:
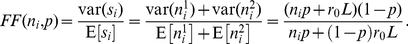
(24)


Note that, for the mean value 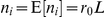, the Fano factor simplifies to 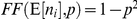. When 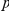 decreases from 1 to 0, 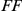 increases continuously from 0 to 1, therefore interpolating from a reliable to a Poisson (highly variable) spike train. Jitters for Model SDJ do not affect the spike count and thus neither the Fano factor.

For model R, the Fano factor for the spike count of any input is 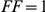, since each spike train is generated using a single inhomogeneous Poisson process.

Note that these Fano factors correspond to continuous time. The finite time step used in discrete-time simulations has an effect, which we minimize by taking sufficiently small time steps.

### Convergence index

**Figure 3:**
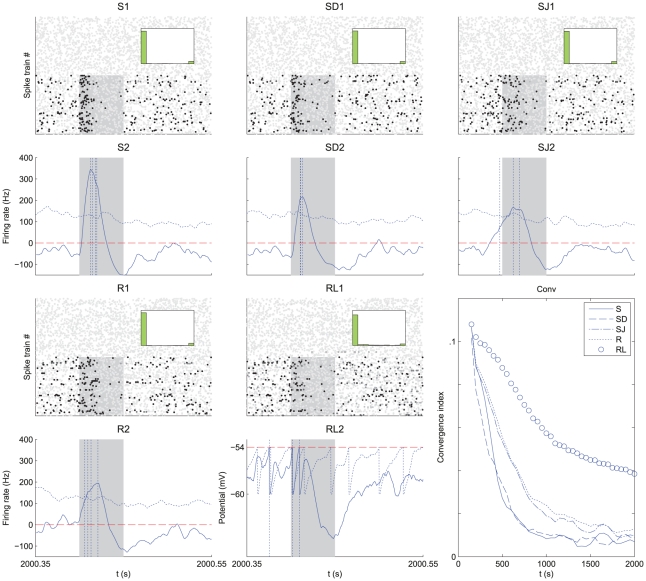
Emerged input selectivity after learning (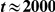 s). Comparison between a trained Poisson neuron for the different pattern types in Fig. 1: (S) model S with 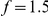 Hz, 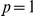, and 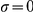 ms, (SD) model S with 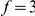 Hz, 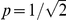, and 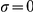 ms, (SJ) model S with 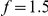 Hz, 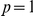, and 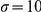 ms and (R) model R with 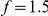 Hz and width 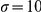 ms; and (RL) a trained LIF neuron with the model R. All patterns have the same latencies 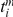. See Text S1 Section S3.2 for details about the parameters. Top panels (label 1): Input raster plots for 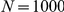 afferents. Darker grey dots indicate stronger weights for synapses whose EPSPs significantly contribute to variations of the soma potential. Again each pattern presentation is indicated by a grey rectangle. In all plots, the cluster of black dots at the beginning of the pattern presentation indicates that STDP has potentiated synapses corresponding to early spikes in the pattern. All non-pattern synapses have been almost completely depressed. The insets display the weight histogram at the end of the learning epoch: the distribution is bimodal with about 70 out of 1,000 potentiated synapses. Bottom panels (label 2): Evolution of the lumped EPSPs (solid curve), namely the contribution to 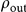 in the rhs of Equation (4) without the positive part for Poisson neurons and 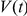 in Equation (S34) for the LIF neuron. The horizontal dashed line indicates the “threshold”: zero for Poisson neurons (under which no spike is emitted), and 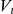 for the LIF neuron; the vertical dashed lines the output spikes. The dotted curves represent the lumped EPSPs before training. (Conv) Plot of the convergence index defined in Equation (24) as a function of time for all models. A lower value indicates a bimodal distribution of the weights at the bounds, to evaluate the learning progression.

In Fig. 3Conv, we use the convergence index:
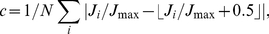
(25)where 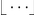 denotes the floor function and 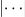 the absolute value. This index is positive and vanishes when all synapses are either maximally reinforced or completely depressed (i.e. bimodal distribution). It was calculated every 50 s and a running average of the last four data points was used in order to remove noise in the plot.

### Mutual information

We use information theory to quantify how good the postsynaptic neuron is at detecting the beginning pattern after convergence (section “General case of an arbitrary pattern”). Detection is considered to be successful when the neuron fires at least two times at the beginning of the stimulus presentation. Specifically, we discretize time into 25 ms bins. Each of those bins could either correspond to the first 25 ms of the pattern (or stimulus), case referred to as 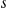, or not (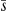), and could contain at least 2 postsynaptic spikes (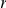) or not (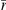). Note that for the LIF neuron the time window considered for counting spikes was actually shifted 10 ms backward since, because of 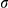
_,_ the potential rise actually starts before the theoretical start of the pattern presentation, which may shift backward the postsynaptic spikes signaling the pattern. The Poisson neuron does not require this because the rate increases mostly during the pattern presentation, and eventual preceding spikes have no influence. For both neurons the mutual information between the postsynaptic response and the presence of the stimulus is:
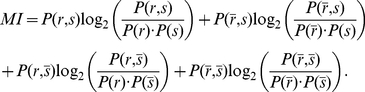
(26)


Note that in signal detection terms, the first term corresponds to “hits”, the second to “misses”, the third to “false alarms”, and the last one to “correct rejections”. A perfect detector would lead to 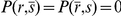, 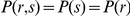 and 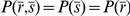. An upper bound on the mutual information is given by:
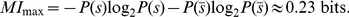
(27)Details can be found in Equation (S31) in Text S1.

## Results

Throughout this paper, we consider a single pattern that is repeatedly presented to an unknown subset of 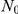 among 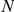 excitatory afferent (or input) plastic synapses that stimulate a single neuron (Fig. 1Schema). We use different models for the generation of input spike trains, as detailed in Materials and Methods: spike patterns where the spike times are fixed (model S) or exhibit jitter compared to a reference (model SJ); and patterns that consist of fast covarying rate functions (model R).

We first show that the repeating pattern presentations determine a specific spike-time cross-correlation structure between afferents. Despite qualitative differences between spike and rate patterns, we found a common expression for their corresponding correlograms. Then, we examine how STDP can capture such signatures and select some inputs involved in the pattern activity, at the expense of all other (pattern and non-pattern) afferents. We finally evaluate the neuronal selectivity to the learned pattern, depending on the pattern, STDP and neuronal parameters.

### Spike-time correlograms as the signature of a repeating pattern

We focus on the situation where the pattern is difficult to learn and detect. Namely, relatively short duration 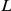 and low presentation frequency 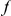 imply that the mean firing rate (averaged over hundreds of milliseconds) is roughly the same across all afferents irrespective of the pattern presentations (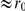, which further requires 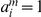 for model R). For the example of model S, our choice of parameters implies that the discrepancies in the mean rate across inputs typically correspond to 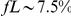, synonymous with very low signal-to-noise ratio as far as learning is concerned. In this case, neuronal specialization cannot be achieved relying on a simple rate difference.

The key to learn such patterns consists in extracting information contained at a fine timescale: the repetitive pattern presentations induce spike-time correlations between pattern inputs (see Materials and Methods). Using our theoretical framework, we can derive a common equation for the cross-covariance 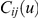 between inputs 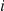 and 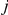 that is valid for all models S (including SJ) and R:
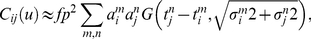
(28)which is a sum of Gaussian kernel functions 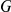 defined in Equation (15) centered around the latencies 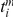 that determine the pattern. The (time-averaged) cross-correlogram in Fig. 1 (right panels) corresponding to 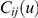 contains information about the spike timing (model S) or spike distribution (model R) within the pattern, as illustrated by Fig. 1. Both the relative latency positions 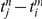 and temporal resolution 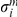 of the pattern determine peaks in the correlogram. For model SD where pattern spikes occur with a given probability 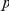 at each presentation, it is clear from Equation (1) that a pattern with a frequency 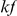 (with 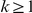) and occurrence probability 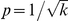 has the same correlogram as the same pattern with reliable spikes (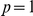) and frequency 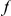 (Fig. 1S2 and SD2 with 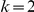). This means that non reliability of firing can be compensated by more frequent repetition, as far as spike-time correlations are concerned.

For model SD, the Fano factor of the spike count scales between 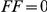 (reliable) for 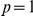 to 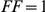 (Poisson) for 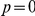 (see Equation (24) in Materials and Methods) and the influence of such a variability on learning will be examined. On the other hand, model R always gives 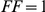 as any Poisson process does. For both models SJ and R, the temporal precision can be varied through 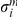. In particular, with a same value for 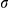, they may only differ by the Fano factor. Fig. 1SJ2 and 1R2 show that Gaussian jitters for model S and Gaussian-peaked rate modulations for model R lead to similar correlograms when they have the same temporal accuracy 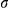. By comparing results for these models, we will assess the effect of the Fano factor on both learning and detection afterwards.

### Weight specialization induced by temporally Hebbian STDP

Now we examine how this spiking information induced by pattern presentation can be captured by STDP. Our theoretical analysis is based on the additive STDP rule and Poisson neuron model, which allow the prediction of the weight specialization induced by STDP [4], [27]. In our phenomenological model of synaptic plasticity, STDP is described by a temporal learning window (Fig. 2A). The Poisson neuron linearly sums excitatory postsynaptic potentials (EPSPs) resulting from each incoming spike to determine the soma potential, which is used as an instantaneous rate function to generate an output spike train. The theoretical predictions will be verified numerically using both Poisson and leaky integrate-and-fire (LIF) neurons, and the effect of weight dependence in the STDP rule will be also examined. Details are provided in Materials and Methods.

The first stochastic moment for the plastic weight 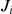 follows a differential equation that is of the form:
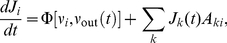
(29)cf. Materials and Methods before Equation (10). Here 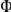 is a function of the mean (time-averaged) firing rates 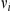 and 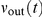 of the 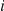-th afferent and neuron, respectively; see Equation (6) for the detailed expression. The coefficients 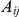 of matrix 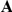 defined in Equation (11) involve the spike-time cross-correlations between input spike trains 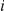 and 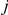. Note that for the pattern models S and R, the input firing rates and spike-time cross-correlograms are time invariant; the output neuronal firing rate only evolves as the consequence of the learning process.

This dichotomy between mean firing rates on the one hand and spike-time correlation coefficients on the other hand highlights the separation of timescales in representing the information contained in spike trains [4], [27]. In our framework, the firing rates 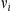 and 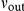 are low-pass filtered variables, but spiking information at a short timescale is still contained in the time-averaged spike-time correlations 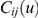 through the time variable 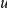 (see Equation (9) in Materials and Methods). Consequently, the weight evolution can be analyzed as a double dynamics that is a mixture of:

- a homeostatic equilibrium that stabilizes the mean incoming weight (over all inputs) and relates to the first term in Equation (29);
- a specialization by competition between individual weights related to the spike-time correlations via the second term in Equation (29).

A typical example is illustrated in Fig. S1 in Text S1. Specialization leads to the potentiation of some weights at the expense of others, then the neuron behaves as a coincidence detector for the selected inputs. The weight selection can be predicted via the matrix 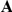, which is tractable when using the Poisson neuron model. Because of the similarity between the correlation structures that they induce, cf. Equation (28), a common expression for the coefficients 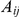 that appear in Equation (29) can be derived both for models S and R. It relies on the difference between the latencies 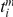 and 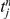 of inputs 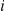 and 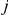, namely
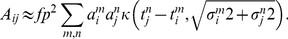
(30)


Model S corresponds to 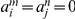 (in addition to 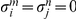 for models S and SD, but not SJ) and model R to 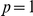. The kernel function 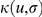 is defined by
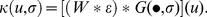
(31)


Note that 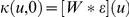, which is represented in Fig. 2A. As shown in Fig. 2B, the more spread the Gaussians are (viz. larger value for 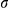), the smoother and smaller the function 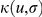 is as a function of the time difference 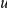.

From the Equation (31), it can be seen that the STDP effects are impaired if the STDP time constants are much smaller than those of the PSP response. This is not the case in the brain (and in our simulations), where all these constants are in the 10–30 ms range.

The spectral analysis mentioned above describes how an initially homogeneous weight distribution will begin to split as a result of STDP. The strength of cross-covariances between pattern inputs reflects their tendency to drive the firing of output spikes. When some weights become stronger compared to the remainder, this driving effect becomes stronger. It follows that the cross-covariances between the potentiated inputs and the postsynaptic neuron increase in a causal manner, in turn inducing further (and stronger for additive STDP) potentiation. This self-reinforcing mechanism (described in more detail in Materials and Methods) leads to a clear potentiation of some pattern inputs, until they either saturate at the upper bound (additive STDP) or reach an equilibrium value (weight-dependent STDP).

### Synapses with earliest spikes are selected in general

Competition between weights 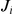 leads to the reinforcement of those with stronger correlation term, so the rule of thumb is that inputs 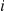 with larger coefficients 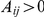 (for all indices 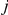) will be selected by STDP. Because 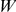 is roughly antisymmetric and we do not consider slow synapses, 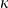 in Equation (30) is such that negative arguments contribute positively to the sum in coefficients 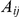. It follows that pattern synapses with early spikes tend to be selected when the spike density is somehow constant for all pattern inputs (see Materials and Methods). When some strong inputs start driving the neuronal firing (as mentioned above), other pattern inputs corresponding to earlier spikes also tend to be reinforced for temporally Hebbian STDP, which further favors inputs with early spikes until the weight strengthening finally stabilizes. This is illustrated for models S and R by simulations using Poisson neurons in Fig. 3S1, SD1, SJ1 and R1 (black clusters). Non-pattern synapses have also been completely depressed by STDP.

While keeping the output neuronal firing rate low on average (due to the homeostatic equilibrium), the potentiated synapses (due to weight competition) ensure a significant increase of the membrane potential (solid curve) at the beginning of each pattern presentation. This usually causes early postsynaptic spikes (vertical dashed lines) that can be used for fast pattern detection (quantification with mutual information will be discussed later). The trained neuron is thus selective to the quasi-simultaneous arrival of the earliest pattern spikes, and can serve as “earliest predictor” of the subsequent spike events, at the risk of triggering a false alarm if these subsequent events do not occur, but with the benefit of being very reactive (to learn the full pattern, several neurons in competition can be used [37]). Comparatively, the membrane potential before training (dotted curve) is similar during and outside pattern presentation.

Model S with 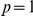, 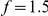 Hz (and 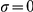 ms), or with 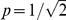, 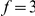 Hz (and still 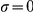 ms) have the same correlation structure (Fig. 1S2 and SD2). Therefore, despite different Fano factors, they lead to the same final weights (Fig. 3S1 and SD1 insets), at approximately the same speed (Fig. 3Conv). However, after learning the increase of the summed EPSPs (solid curve) is stronger in the first case (Fig. 3S2) than in the second (Fig. 3SD2) because of the missing spikes.

Likewise, model SJ with 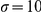 ms (and 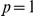, 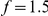 Hz) and model R with 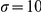 ms (and still 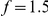 Hz) have similar correlation structures (Fig. 1 SJ2 and R2) and thus lead to the same final weights (Fig. 3SJ1 and R1 insets). Here too, they have approximately the same learning speed (Fig. 3Conv) despite the difference in their Fano factors. The use of 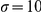 ms induces a more spread rise of EPSPs, comparable in both cases (Fig. 3SJ2 and R2). Indeed, with model R the deviations from the mean spike counts at each pattern presentation tend to compensate across the n≈70 selected afferents (with Poisson processes like here, the total spike count's coefficient of variation decreases in 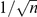). Since the EPSP rise is more spread for model SJ and R than with model S, the detection performance is poorer, but it remains acceptable, even with a decision rule based only on the neuron's spiking output (this will be discussed later).

In our simulations spike count variability (as measured by the Fano factor 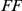) does not slow down the learning, whereas spiking temporal imprecision (related to 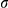) does. In terms of convergence speed, we have S∼SD>SJ∼R (see Fig. 3Conv).

A LIF neuron trained with model R (Fig. 3RL) also selects pattern synapses with early spikes, which results here in two postsynaptic spikes each time the pattern is presented. With our choice of parameters learning was found to be slower for the LIF neuron (Fig. 3Conv). Note that, for model S with other parameters, selectivity can emerge in a few tens of pattern presentation [9]. The fact that the LIF neuron behaves similarly to the Poisson neuron justifies a posteriori the use of the latter for convenience in the theoretical analysis. In a regime where the LIF neuron is sensitive to volleys of almost coincident spikes, only the inputs with the very first spikes may remain potentiated at the end of the learning epoch, whereas the synapses corresponding to later spikes are depressed [9]. However, a thorough discussion of these effects is beyond the scope of the present paper. Here we focus on the Poisson neuron, for which new analytical results are presented below. Extensive numerical studies with the LIF neuron can be found in previous work [9], [37], [38].

### Influence of the spike distribution within the pattern

**Figure 4:**
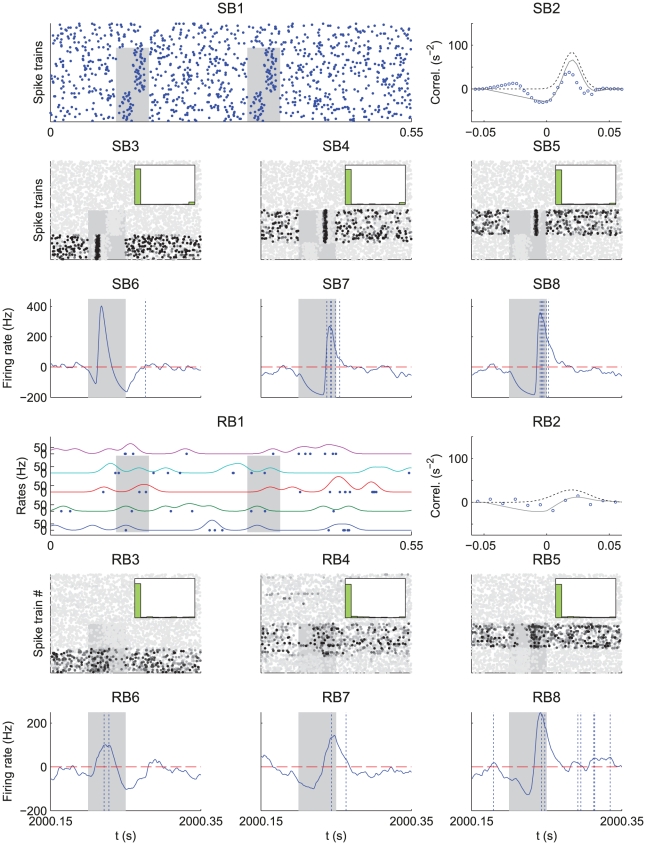
Influence of the spike distribution within the pattern. Comparison between a spike pattern of model SB (SB panels) and a rate-modulated pattern of Model RB (RB panels), both having a bimodal distribution of latencies. (SB1) Model SB. The plot displays 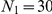 afferents that fire with a mean latency 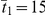 ms with a spread 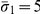 ms around this latency, and 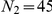 afferents that fire with a mean latency 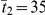 ms and same spread 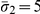 ms. (SB2) The average cross-correlogram over the respective groups (circles) corresponds to Equation (16). (RB1) Model RB. The plot displays two afferents of each group. In each group all the afferents have the same pattern rate function: Gaussians centered respectively on 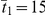 ms and 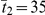 ms with width 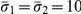 ms and amplitudes 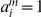. (RB2) The cross-correlogram between two afferents in distinct groups exhibits a peak at 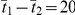 ms. Plots SB3–8 and RB3–8 are similar to those in Fig. 3. (SB3,SB6 and RB3,RB6) 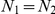 and 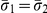: the earliest cluster is selected. (SB4,SB7 and RB4,RB7) 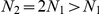 and 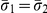: the latest cluster is selected. (SB5,SB8 and RB5,RB8) 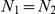 and 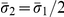: the latest cluster is selected.

The above-mentioned rule of thumb that inputs with early spikes are favored does not hold for all patterns. To illustrate how the spike distribution among pattern inputs affects the weight evolution, we use a specific configuration of models S and R, where pattern afferents are partitioned into two groups (or populations) with respective numbers of inputs 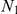 and 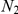. More precisely, this bimodal distribution of input latencies is such that the afferents of group 1 tend to fire before those in group 2:

- Model SB: bimodal spike pattern (Fig. 4SB). Each afferent 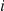 belonging to group 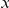 (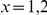) has only one pattern spike corresponding to latency 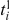, which is randomly drawn from a Gaussian distribution with mean 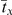 and spread width 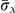. Inputs in group 1 tend to arrive before those in group 2: 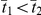. We also set 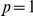 and 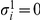 (no jitter).
- Model RB: bimodal rate-modulated pattern (Fig. 4RB). Within each group, all afferents have the same Gaussian rate function centered on 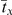 (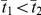) with spread width 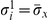 for input 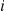 in group 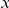; amplitudes are unitary (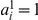).

The overline indicates group variables. In this way, we control the crucial parameters that determine the clustering of spikes within the early and late groups, namely their sizes and temporal resolutions, whose effect will be assessed against the difference between their latencies.

In terms of population averages, both models have the same expression for the input spike-time correlations given in Equations (16) and (17) with respective mean latency 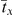 and temporal spread 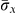, as illustrated in Fig. 4SB2 and 4RB2. This illustrates another connection between these two pattern models despite their different spike generation mechanisms. Note that, for model R, the amplitudes 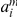 and the number of inputs with clustered pattern spikes (e.g. group size for model RB) plays a similar role.

We consider the situation where the inputs from the first group fire sufficiently early compared to the second group (say 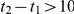 ms). Our framework allows us to study the effect of the pattern parameters on the resulting competition between the two groups; detailed calculations are provided in Text S1 (Section S2.2).When both groups have similar size (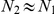) and spread width (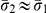), STDP tends to select the first group in agreement to the previous section, see Fig. 4SB3,6 and 4RB3,6. However, when the second group is more populated (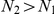), STDP preferably selects the late group as shown in Fig. 4SB4,7 and 4RB4,7. Now, when the second group has a narrower spread (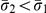), while both groups have comparable size (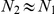), STDP tends to select the second group as shown in Fig. 4SB5,8 and 4RB5,8. This extends previous results on the effect of correlation spread for two input groups that have no correlation between them [39]. Simulations with models SB and RB exhibit similar trends, in agreement with the resemblance between their (population averaged) spike-time correlograms.

In summary, potentiation of synapses corresponding to early pattern spikes competes with another trend that favors densely populated and narrow spike clusters, irrespective of the spike generation type within the pattern. Note that this does not affect the success of pattern detection, but only its timing.

### General case of an arbitrary pattern

**Figure 5:**
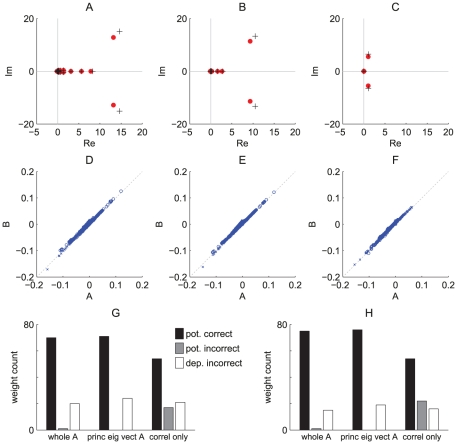
Theoretical prediction of the weight specialization. Comparison between the matrices 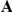 and 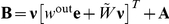 in Equation (10) for a given pattern of model SJ (or its equivalent of model R) using various values for 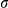: (AD) 0 ms, (BE) 10 ms and (CF) 30 ms. (ABC) Spectra of matrices 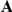 (pluses) and 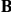 (dots). (DEF) Comparison between the principal eigenvectors (one of the complex conjugated pair) of matrices 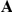 (x-axis) and 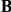 (y-axis). The positive and imaginary parts of the coordinates for are represented by crosses and circles, respectively. The diagonal indicates perfect alignment. For (G) Model SJ and (H) model R, comparison between simulated (Fig. 3) and predicted weight evolution. After 2000 s, weights are labeled as potentiated if above 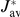 and depressed otherwise; both simulations with models S and R exhibit around 70 potentiated and 930 depressed weights. The black and grey histograms count the number of potentiated weights that are correctly and wrongly predicted, respectively; the white histogram the depressed weights incorrectly predicted. The prediction ‘whole A’ are obtained by simulating the differential equation (10) with the whole matrix 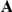 in 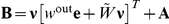, whereas ‘princ eig vect A’ only involve the two principal complex-conjugated eigenvectors of 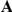, cf. (B). The prediction ‘correl only’ uses a projection the principal eigenvectors of 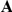 to analytically predict the potentiated weights, which ignores rate effects.

Now we go back to the case of a general pattern that has arbitrary latencies 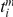. We examine how the trends revealed by the analytical study of models SB and RB adapt here. A complete description of the weight evolution involves the spectrum of matrix 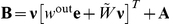, as Equation (28) can be rewritten as a linear differential matrix equation of the form 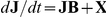 (see Equation (10) in Materials and Methods). The spectrum of this matrix (circles) is represented in Fig. 5ABC for a pattern of model R: most eigenvalues are close to those of 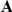 (pluses), which can thus be used to predict the weight evolution; the large negative eigenvalue of matrix 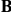 roughly equal to 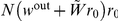 and associated with the homeostatic equilibrium is not displayed there for clarity. Note that eigenvalues may be complex numbers. Likewise, the dominant left eigenvector(s) that determine the weight specialization are similar for both matrices (Fig. 5DEF). As shown in Fig. 5GH, the weight evolution can be satisfactorily predicted using either the whole matrix 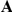 (label ‘whole A’) or its principal eigenvectors (label ‘princ eig vect A’) both for models S and R. Note that our predictions slightly overestimate the number of potentiated weights here (as in Fig. S1, in Text S1). Neglecting rate effects, more than 80% of the potentiated weight can be predicted (label ‘A only’), meaning that spike-time correlations dominate.

**Figure 6:**
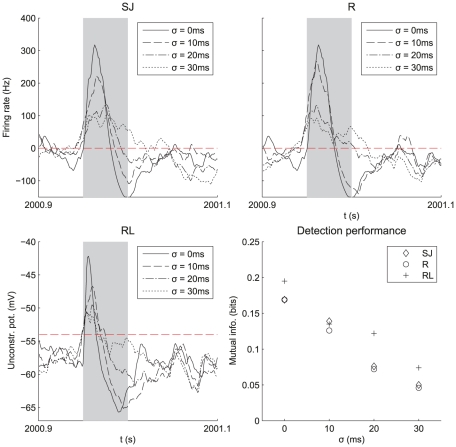
Effect of the jitter 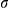 for model SJ, and the spread width 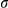 for model R on learning and detection. Model R is also tested on a LIF neuron (panel RL). The plots are similar to Fig. 3, except for the LIF neuron: the unconstrained postsynaptic soma potential that is plotted ignores the threshold (horizontal dashed line). When the pattern is presented (grey area), the soma potential rise is stronger for small 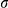, synonymous with more robust selectivity, even though detection does not completely collapse for larger values. To quantify the detection quality we used mutual information between pattern presentations and postsynaptic spikes (see Equation (26) in Materials and Methods): the latter decreases with 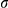. The LIF neuron is on average slightly more reliable than the Poisson neuron (here perfect detection corresponds to mutual information equal to 0.23 bits).

In a similar manner to the simpler model RB when a pattern of model R has more spread rate peaks, the elements of 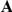 go to zero (Fig. 5ABC) as the function 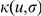 becomes flatter when 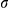 increases (Fig. 2B). Consequently, the weight specialization weakens, which significantly decreases the quality of detection, as estimated by the mutual information defined in Equation (25) and plotted in Fig. 6. With the same spread width, Gaussian jitters (Fig. 6SJ) and Gaussian rate peaks (Fig. 6R) give similar performance: because sufficiently many inputs are used spiking variability of model R (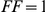 within the pattern presentation) hardly impairs the detection w.r.t. Model SJ with (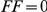 for 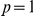). Together with results comparing models SB and RB in Fig. 4, this supports our conclusion that the weight evolution induced by STDP is mainly determined by the input spike-time cross-correlograms, and that unreliability in the spike count (as measured by the Fano Factor) has little effect.

**Figure 7:**
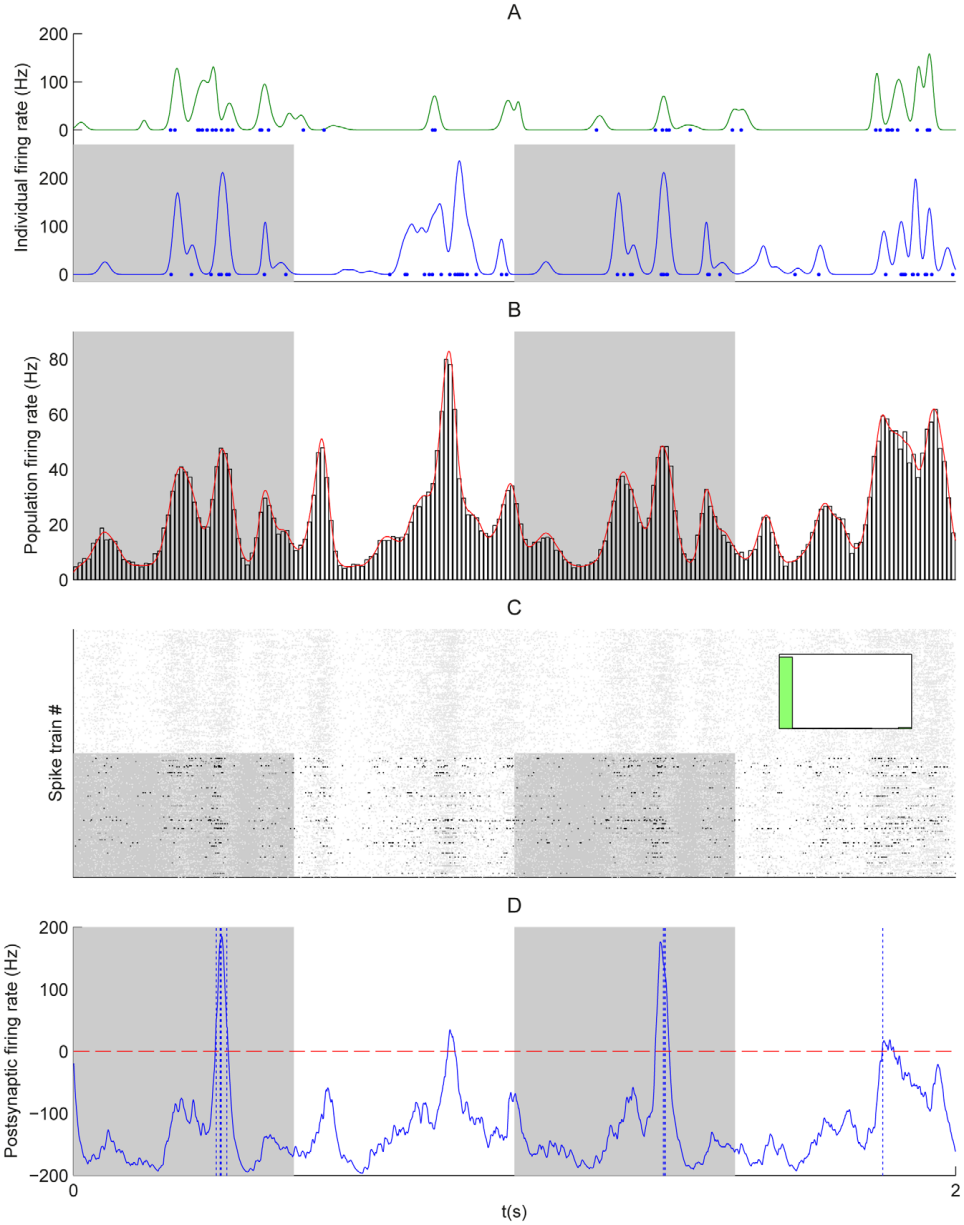
Long pattern of model R with duration 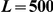 ms and global time-varying spike density. (A) Individual rate functions for a pattern (bottom) and a non-pattern (top) input. During all pattern presentations (grey areas), the pattern input exhibits the same rate modulations (similar plot to Fig. 1R1). The width of rate peaks is randomly chosen around 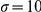 ms and the amplitudes (e.g. 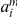) are multiplied by a common temporal envelope (see text). (B) Population-averaged spike histogram (bars) and theoretical firing rate (solid line, average of individual rate functions of (A)). (C and D) Input raster plot and lumped EPSP response after learning (similar to Fig. 3). The black cluster in (C) indicates that the third population peak in the pattern in (B) has been selected. The inset displays the bimodal final weight distribution and shows that only a few weights were potentiated. While remaining sensitive to the mean input spike rate in (B), the postsynaptic neuron is above all selective to the third pattern peak for the two presentations displayed in (D).

Now, as an illustration of an arbitrary pattern that mimics real PSTHs, we consider a longer rate-modulated pattern (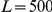 ms). We assume that the inputs are modulated by some global strength (e.g. image or movie contrast, sound volume), while individual rates encode local features. For this purpose, the rate functions are multiplied both for pattern and non-pattern inputs by a common envelope. The latter corresponds to superimposed 20-ms spread peaks distributed in time with a homogeneous Poisson process at 20 Hz, as illustrated in Fig. 7AB. For all inputs, the envelope is repeated identically during all pattern presentations. In our simulation, STDP selects a single cluster of rate peaks in the pattern (e.g. third peak in Fig. 7CD) and mainly reinforces inputs corresponding to that “population” peak. In agreement to the results for the simpler model RB, STDP favors clustered, high-amplitude and narrow modulations. Interestingly, even though the population firing rate in Fig. 7B may be much larger outside than during pattern presentations, the neuron fires almost only during the presentations (Fig. 7D).

To further evaluate the relative strengths of the mean input firing rates and spike-time correlations induced by the pattern, we also simulated a pattern with a lower firing rate than the noisy activity surrounding it: 15 Hz vs. 25 Hz, respectively. The output neuron could still be trained successfully, although not as well as for the control case of 20 Hz vs. 20 Hz (not shown here).

These results show that STDP can learn non-flat rate-modulated patterns relying on the same competition between afferent weights as described above for the more difficult case of quasi constant input firing rate. Realistic PSTHs can thus be learned provided sufficiently many inputs exhibit significant rate modulations on a “fast” time scale, namely ranging up to 10–20 ms with our parameters. In this case, correlation effects dominate the weight dynamics.

### Influence of the STDP and neuronal parameters

Finally, we examine the influence of the STDP and neuronal parameters on both learning and detection afterwards for a typical pattern of model R (similar results were obtained with model S). To estimate the quality of detection, we evaluate the neuronal response after training averaged over 10 pattern presentations (in order to remove noise and display clear trends). We keep in mind that the goal is detection of *each* pattern presentation by the neuron, though.

**Figure 8:**
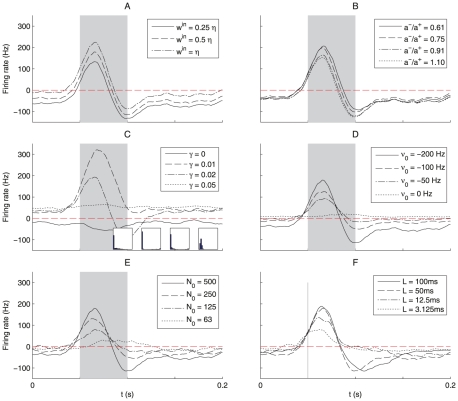
Parametric study of the detection robustness. In each panel we have varied one parameter, all others remaining identical to the baseline choice (except for 8C). The plotted postsynaptic response to a given pattern of model R (after learning) is averaged over 10 presentations (grey area). The plots are similar to the bottom panels in Fig. 3. (A) Influence of 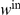: a higher value enforces a higher mean firing rate on the neuron, leading to a weaker selectivity. (B) Influence of the 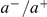 ratio: the bigger this ratio is, the more biased towards depression STDP is (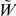 becomes more negative). Due to the stabilizing effect of the homeostatic rate-based terms 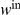 and 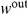, the effect of this ratio is weak (while keeping 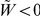). In contrast, without 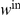 and 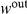, the ratio 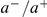 needs to be fine-tuned to obtain successful training [9] (C) Influence of the strength 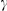 in weight-dependent STDP: the parameter 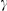 scales between additive (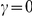) and multiplicative (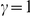) [6], without homeostatic terms (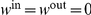). Slightly-multiplicative STDP (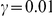) leads to good detection performance, which collapses for higher values. (D) Influence of 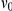 (cf. Poisson neuron model): a less negative value leads to a lower selectivity, and it completely collapses without inhibition (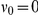). (E) Influence of the number 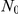 of pattern inputs: detection remains acceptable down to 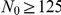, corresponding to 12.5% of the total number 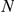 of inputs. (F) Influence of the pattern duration 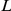: even though small 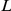 decreases the performance, the pattern is still successfully detected; for 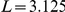 ms, the configuration is almost equivalent to a synchronized input group [4] However, STDP performs better for longer patterns and the beginning of all patterns has been learned here.

In our model, the mean output firing rate of the neuron is constrained by STDP close to an equilibrium value 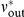 that depends on the rate-based contributions 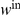 and 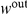, the ratio LTP/LTD (related to 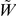), and the background excitation/inhibition 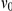 (cf. Equation (4)). For a good detection performance, in other words minimizing false alarms, 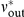 in Equation (20) must be kept low, which can be achieved using a small value for 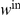, as illustrated in Fig. 8A. However, such a small value also decreases the magnitude of the response to the pattern. The use of 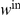 and 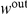 prevents the pitfalls where all synapses become depressed, leading to a quiescent neuron, or on the contrary maximally reinforced, leading to a very active non selective neuron. They also allow the use of asymmetric STDP where overall depression is stronger than the overall potentiation (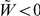), relieving the requirement to tune depression only slightly higher than potentiation [9], as illustrated in Fig. 8B where the ratio between depression and potentiation for STDP hardly affects the detection performance. We also looked at the effects of weight dependence using the model proposed in [6], for which a parameter 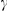 scales between additive (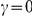) and multiplicative (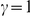) STDP (Fig. 8C). These simulations were done without the homeostatic terms 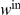 and 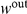, aiming to show that learning is still possible without them. However, we had to fine tune some parameters: 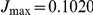 (vs 0.1215 in the baseline simulation, cf. Text S1 Section S3.2), 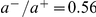 (vs 0.82 in the baseline). Convergence took about 4000 s (vs 2000 s in the baseline). With these values learning fails with pure additive STDP (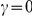), leads to good detection performance with slightly multiplicative STDP (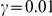). This performance decreases with more multiplicative STDP (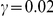), and collapses with 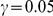, which leads to a unimodal final weight distribution. In comparison, additive STDP, also with 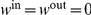, could hardly learn and detect patterns of type SJ with jitters larger than 2 ms [9]. Small values for 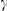 in Fig. 8C ensure both homeostasis and strong competition for weight-dependent STDP [33], which results in good detection for a jitter of 10 ms.

Inhibitory background activity (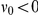) is used to obtain an equilibrium with both a low mean firing rate 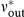 and a positive mean input weight 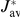 (see Equations (20) and (19)). As mentioned above, the first ensures that the trained Poisson neuron has a low number of false alarms. The second leads to successful detection with sufficiently many potentiated pattern inputs that imply a significant increase of the soma potential during each pattern presentation. In contrast, the case 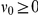 corresponds to a noisy neuron and the pattern response is then not so strong compared to 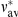, weakening detection as illustrated in Fig. 8D (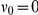). In this case, inhibition helps to increase the sensitivity of the neuron to correlated inputs, both for learning and detection. This effect, together with that of 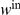, is predicted by the analytical evaluation of the mutual information in Text S1 (Section S2.3).

We also verified the robustness of our scheme with respect to the number of pattern afferents 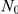. So far, we have used 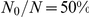, which ensured that STDP could choose among many afferent candidates. Decreasing 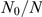 weakens the neuronal response, but down to as few as 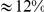, the detection remains acceptable; only for 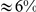 the performance collapses (Fig. 8E). Successful detection can be achieved provided there are sufficiently many pattern inputs such that the potentiation of “half” of them (while depressing the others) leads to a significant response of the soma potential.

Last, Fig. 8F examines pattern of various durations 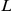 (between 3 and 100 ms). When 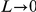, the pattern inputs behave as a narrowly correlated group which is selected by STDP [4], but, not sufficiently many inputs have a rate peak in the pattern, so performance decreases. Increasing 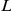 beyond the time scale of STDP does not significantly change detection, since only a portion of the pattern is learned (e.g. the beginning in Fig. 8F). Patterns of duration 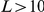 ms fully use the temporal (approximate) antisymmetry of the STDP learning window 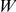.

Taken together, these results show that the proposed STDP-based pattern learning/recognition mechanism works for a broad range of parameters. It also works similarly for Poisson and LIF neurons, which supports the theory that the proposed learning scheme relies on the STDP qualitative properties, namely the temporal antisymmetry of the learning window 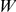, rather than on a precise quantitative configuration or neuronal model.

## Discussion

The contribution of this paper is twofold. First, we have demonstrated how an additive-like STDP leading to strong competition between afferents can train a neuron in an unsupervised fashion to detect not only spike patterns but, more surprisingly, rate-modulated patterns resembling typical PSTHs. Second, we have shown that despite distinct variability (as measured by the Fano factor) but provided they have the same temporal precision, both spike and rate-modulated patterns are equivalent from the point of view of STDP. Both induce similar spike-time correlation structures, hence similar neuronal specializations. Altogether, our results indicate that temporal inaccuracy (up to 10–20 ms), and Poisson-like firing variability (both for the inputs and the postsynaptic neuron) are not an obstacle to robust learning and efficient single-trial detection afterwards by a single neuron.

### Summary of results

A previous simulation study showed that a repeating arbitrary spatiotemporal spike pattern (model S) hidden in continuous equally dense distractor spike trains can be robustly detected and learned by a single neuron equipped with STDP [9] Here we have demonstrated that these results extend to the case of patterns of temporally modulated instantaneous firing rates (model R), from which the spikes are generated at each presentation, e.g., through an inhomogeneous Poisson process. To gain analytical insight, we developed a theoretical framework, which extends previous studies that showed how STDP favors correlated inputs [4], [3], [7], [6]. We confirmed that Hebbian STDP (cf. Fig. 2A) tends to favor synapses corresponding to early spikes in the pattern, generalizing previous results on spike patterns [9]. An interesting corollary is that the neuronal response thus becomes faster presentation after presentation. However, this rule is not general when considering specific spike distributions within the pattern, both for spike and rate-modulated patterns. As summarized in Fig. 4, inhomogeneities of the spike density also play an important role in the selection process, with a preference for densest and narrowest peaks, similar to spike “waves” for synchrony detection. In any case, what is important for successful detection is that some “nearly” coincident pattern inputs are strongly potentiated compared to the others. Whether the selected inputs correspond to an early part of the pattern or not changes only the response timing.

These results are robust and hold for a broad range of STDP, neuronal and pattern parameters (see Fig. 8). The scheme proposed here draws on the approximate temporal antisymmetry of Hebbian STDP and its timescale. Changing the details of the STDP learning window (see Fig. 2A) does not compromise the detection, even though the learning dynamics is quantitatively affected. A larger time constant for STDP can be used to learn patterns with larger temporal spreads than those considered here. Even when input firing rates significantly vary over time (cf. Fig. 7) or are inhomogeneous among inputs, spike effects tend to dominate rate effects in the learning dynamics to determine the neuronal specialization, provided sufficiently many inputs are involved. Similar results can be expected for general input spike trains, provided the firing rates and spike-time correlations are well defined (cf. Equations (6) and (9)).

In the brain, homeostatic synaptic scaling mechanisms ensure that firing rates remain in a suitable range [29]. As in previous studies [4], [39], [27], we modeled those mechanisms with two additive terms occurring whenever an input spike is received (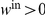) or an output spike is emitted (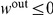). However, even though they increase the learning robustness, simulations show that those terms are not required for its success (Fig. 8C, [9]).

Provided a single pattern keeps being presented, the emerged weight structure is preserved. However, changing the pattern presentation may cause the neuron to forget its specialization. For most weight-dependent models that produce a unimodal (unspecialized) weight distribution for uncorrelated inputs, the structure will be forgotten when the presentation frequency is too low [33]. Additive-like STDP rules can preserve a bimodal weight distribution over a long period even though inputs become uncorrelated [9], although this behavior is somewhat parameter dependent [27], [40]. When several patterns are presented, they compete to determine the emerging weight structure [37] and those with the strongest correlations dominate this competition. If one does not clearly dominate the others, the STDP-induced noise (due to weights jumps) may cause the neuron to switch its specialization between patterns of comparable correlation strength. This happens, for example, when the learning rate is sufficiently large [41].

### Implications for neuroscience

One can distinguish two kinds of response variability, or lack thereof: reliability and precision [42]. When a neuron fires approximately the same number of spikes on each trial, it is said to be reliable, whereas when the spikes occur almost at the same time across trials it is said to be precise. Our study demonstrates that STDP-based pattern learning needs a precision of 10–20 ms, whereas it is almost insensitive to a lack of reliability (see Section S1.6 and Fig. S2 and S3 in Text S1 for the case 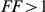), provided the patterns involve at least ∼100 afferents, which is very probable as a typical cortical neuron has about 10,000 afferents. In particular, it can cope with the Poisson-like variability often observed experimentally [43], [44]. Evidence for neural activity with the required precision abounds, at least in sensory systems exposed to dynamical stimuli [18], [19], [20], [21], [23], [22] and, even more importantly, to naturalistic stimuli [24], [25], [26]. Moreover, for slowly varying or even static stimuli, learning with STDP may be still possible when neural activity oscillations are able to attribute to spikes stimulus-specific preferred phases [38].

Importantly, the mechanism we propose does not need an external time reference such as a stimulus onset. Patterns are learned and recognized is a clock-free system, as they are embedded in random but similar spiking activity. This is possible because STDP only relies on relative timing. A low absolute time precision with respect to the stimulus onset (e.g., estimated by a PSTH) does not preclude far more precise relative latencies, in agreement with experimental observations [45], [46], [47], [25], [48] For example, this is the case with trial-dependent input fluctuations (e.g., correlated noise) that affects spike times similarly.

At a broader level, the rate-modulated patterns considered here can account for a large class of the naturalistic temporal stimuli [17] that a subject experiences in vision, audition, touch or multimodal integration, and we demonstrated that STDP genuinely enables learning and recognition for such patterns. This suggests an important role of this plasticity rule in the development and learning of receptive fields, in perceptual learning, which typically involves many repeated trials [49] and, beyond sensory processing, in other cognitive processes or tasks with fast time scales (tens of ms). Importantly, the relatively low precision and high firing variability considered here correspond to a much weaker assumption than models of spike trains generally used with STDP, and the present results reconcile STDP with experimentally observed spike trains.

### Future work

A recent study has proposed a general scheme for the derivation of a linear-nonlinear Poisson (LNP) cascade model to reproduce the dynamics of a spiking neuron [38] In short, a stochastically firing LNP neuron model describes how the input current is filtered by the synapses and dendrites (linear part), and then transformed to calculate the instantaneous rate (nonlinear part). The Poisson neuron used here is a particular and simple case in the class of LNP models. In particular, we have not considered here with the Poisson neuron a linear filter (described by the PSP kernel 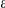) that diverges at the time origin, which is necessary to match the LIF dynamics. Further analysis using the LNP model could help to understand in more depth the general agreement and specific discrepancies between the LIF and the Poisson neurons in our scheme.

For biological realism, a desirable extension of the present framework consists in the learning of a pattern ensemble with a recurrent neural network. This can be achieved with a simple connectivity scheme where afferent connections are trained similarly to the single neuron case: a differentiated specialization can be achieved using lateral inhibition, which prevents neurons from learning the same pattern features [37] In the case of a general recurrent network with plastic synapses, the learning and the neuronal dynamics are intricately coupled and new behavior will certainly appear [50] For a particular network configuration with plastic recurrent synapses, a numerical proof that STDP can successfully learn and retrieve spike patterns (model S) has been given [51] A layered network with both feed-forward and lateral STDP-plastic connections was shown to reliably transmit volleys of almost synchronous spikes by means of delay selection [52] with jitters up to 20 ms. Our results are consistent with that previous numerical study in the sense that STDP tends to select among all incoming connections those corresponding to denser clusters of spikes. This is indeed synonymous with a higher synchronization among the selected inputs, and because neurons within each layer have lateral connections, they tend to synchronize their output spike volley. A recurrent network where STDP operates can also generate spontaneous and repeating patterned activity [53], [54], [55] Our findings also suggest that similar results may be obtained with rate-modulated patterns (model R). Beyond these particular results, the existence and the properties of a mapping between input patterns and output patterns of interconnected neurons whose connections are modified by STDP remains unknown. For example, the ongoing activity in a network as observed in the brain (e.g., due to background inputs) can clearly affects the “signal-to-noise ratio” of pattern activity and should be taken into account. However, the present analysis is a necessary prerequisite to identify some important dynamical ingredients that may allow a network to learn, retrieve and generate a pattern ensemble.

## Supporting Information

Text S1 Detailed calculations about the statistics concerning the pattern inputs (Equations (12), (13), (14), (16) and (17)); the derivation of Equation (24) involving the Fano factor for model SD; details about the homeostatic equilibrium (Equations (18), (19) and (20)); the predicted weight evolution for models SB and RB in Fig. 4; an analytical evaluation the mutual information defined in Equation (26); comments about the previous work in [9]; details about the parameters used in numerical simulations; four supplementary figures that further describe our results.
(DOC)
