# Supplementary material for: STDP Allows Fast Rate-Modulated Coding with Poisson-Like Spike Trains
Source: PLoS Comput Biol. 2011 Oct 27;7(10):e1002231. doi: 10.1371/journal.pcbi.1002231 (PMC3203056; doi:10.1371/journal.pcbi.1002231)
Supplement: Text S1 — Detailed calculations about the statistics concerning the pattern inputs (Equations (12), (13), (14), (16) and (17)); the derivation of Equation (24) involving the Fano factor for model SD; details about the homeostatic equilibrium (Equations (18), (19) and (20)); the predicted weight evolution for models SB and RB in Fig. 4; an analytical evaluation the mutual information defined in Equation (26); comments about the previous work in [9]; details about the parameters used in numerical simulations; four supplementary figures that further describe our results. (DOC) [file pcbi.1002231.s001.doc]

# Supplemental Text S1 - STDP allows fast rate-modulated coding with Poisson-like spike trains

Matthieu Gilson*, Timothée Masquelier* and Etienne Hugues

* These authors equally contributed to this work.

# S1 Complements on input spike train structure

### In this section, we provide detailed calculations that lead to the analytical expressions for the firing rates and spike-time covariances for the different pattern model in the main text, namely Equations (12), (13), (14), (16) and (17). Section S1.6 also provides details on spike patterns with Fano factors larger than 1.

### S1.1 Firing rates for all pattern models

First, we consider a spike pattern with no jitter of model S. For any input $i$, the instantaneous firing rate (or spiking intensity) outside the pattern presentation is constant:

$\left\langle S_{i}\left(t'\right)\right\rangle=r_{0}$.

(S1)

For non-pattern inputs, Equation (S1) is also valid during pattern presentation: therefore, the mean (time-averaged) firing rate defined in Equation (6) in the main textError: Reference source not found is constant and equal to

$\nu_{i}\left(t\right)=\frac{1}{T}\int_{t-T}^{t} \left\langle S_{i}\left(t'\right)\right\rangle\text{d}t'=r_{0}$,

(S2)

which is Equation (12)Error: Reference source not found in the main text.

For pattern inputs with $p=1$, we discriminate periods inside and outside pattern presentation (using the characteristic function $\mathbf{1}_{t'}=1,0$ during each period, respectively):

$$\begin{matrix} \nu_{i}\left(t\right)=\frac{1}{T}\int_{t-T}^{t} \mathbf{1}_{t'}\left\langle S_{i}\left(t'\right)\right\rangle\text{d}t'+\frac{1}{T}\int_{t-T}^{t} \left(1-\mathbf{1}_{t'}\right)\left\langle S_{i}\left(t'\right)\right\rangle\text{d}t'\approx\frac{fT}{T}\int_{0}^{L} \left\langle S_{i}\left(t'\right)\right\rangle\text{d}t'+\left(1-\frac{fT}{T}L\right)r_{0} \\ =r_{0}+f\left(n_{i}-Lr_{0}\right) \end{matrix}$$

(S3)

after a change of variable for pattern presentations ($0\leq t'\leq L$); $n_{i}$ is the number of spikes within the pattern for input $i$. The approximation in Equation (S3) corresponds to neglecting the border effects at $t$ and $t-T$, namely the situation where some pattern presentation exceeds this interval. Jittering does not change this result except for border effects. We ignore the corrections related to potentially overlapping pattern presentation, in this way considered simply Poissonian with intensity $f$. It follows that the mathematical expectation and variance for $\left(1/T\right)\int_{t-T}^{t} \mathbf{1}_{t'}\text{d}t'$ are (to a good approximation) and $fT/T^{2}=f/T$, respectively. This means that, for a sufficiently large $T$, the mean firing rate is quasi-independent of time ($f/T\to0$).

Furthermore, because the pattern spike times are also Poisson-distributed within the presentation time interval $\left[0,L\right]$, the mathematical expectation and variance for the spike counts $n_{i}$ across all inputs $i$ have the same value: $\mathrm{E}\left[n_{i}\right]=\mathrm{var}(n_{i})\approx Lr_{0}$. This gives the variance of the mean firing rates (considered to be time invariant) in Equation (S3) over all inputs with respect to their mean $r_{0}$: $\mathrm{var}(\nu_{i})=f^{2}\mathrm{var}(n_{i})\approx f^{2}Lr_{0}$. It follows that, when the presentation frequency $f$ is small (such that $f^{2}L<<1$ since we use a small value for $L$), the mean firing rates are almost identical for all inputs:

$\nu_{i}\left(t\right)\approx r_{0}$.

(S4)

A similar calculation applies to the case $0<p<1$: the additional spike train that compensates for the “missing” spikes is generated by an additional Poisson spike trains with intensity $\left(1-p\right)r_{0}$. This does not change the mean, but slightly increases the variance, which remains negligible for sufficiently large $T$ and small $f$. See the calculations leading to Equation (24) for the evaluation of the effect of $p$ on the Fano factor in the main text.

Second, we present similar calculations for a general pattern of model R. For any input $i$, the instantaneous firing rate is described by the succession of Gaussian rate peaks centered in times $t_{i}^{m}$:

$$\left\langle S_{i}\left(t\right)\right\rangle=\sum_{m} a_{i}^{m}G\left(t-t_{i}^{m},\sigma_{i}^{m}\right)$$

(S5)

with the normalized Gaussian kernel defined in Equation (15) in the main text, with mean zero and variance $\sigma_{i}^{m}$.

As $\int_{t_{0}-D}^{t_{0}+D} G\left(t'-t_{0},\sigma\right)\text{d}t'\approx1$ for any suitable intermediate duration $\sigma<<D<<T$ (which we assume for all $\sigma_{i}^{m}$), the mean firing rate can be evaluated as

$\nu_{i}\left(t\right)=\frac{1}{T}\int_{t-T}^{t} \left\langle S_{i}\left(t'\right)\right\rangle\text{d}t'\approx\frac{1}{T}\sum_{t-T<t_{i}^{m}<t} a_{i}^{m}$.

(S6)

The approximation here relates to border effects at $t$ and $t-T$, which are negligible for sufficiently large $T$ (same argument as with model S above); firing rates are also quasi time-invariant for model R. Because times $t_{i}^{m}$ are distributed as the spike times for model S, the mean firing rate is roughly equal to $r_{0}$ when choosing $a_{i}^{m}=1$, leading to Equation Error: Reference source not found(12) in the main text.

### S1.2 Spike-time covariance structure for pattern model S

For a pattern of model S, whenever at least one of the inputs $i$ and $j$ is a non-pattern input, we have

$\left\langle S_{i}\left(t'\right)S_{j}\left(t'+u\right)\right\rangle=\left\langle S_{i}\left(t'\right)\right\rangle\left\langle S_{j}\left(t'+u\right)\right\rangle=r_{0}^{2}$,

(S7)

since the two spike trains are probabilistically independent. In this case, $C_{ij}\left(t,u\right)=0$. For the same reason, when at least one of the two pattern inputs is outside the pattern presentation (i.e., either $t'$ or $t'+u$), the expression in Equation (S7) evaluates to the product of the stochastic intensities, which gives $r_{0}^{2}$ when averaged over time. We operate the same dichotomy as in Equation (S3) and obtain

$\frac{1}{T}\int_{t-T}^{t} \left\langle S_{i}\left(t'\right)S_{j}\left(t'+u\right)\right\rangle\text{d}t'\approx\frac{1}{T}\int_{t-T}^{t} \mathbf{1}_{t'}\mathbf{1}_{t'+u}\left\langle S_{i}\left(t'\right)S_{j}\left(t'+u\right)\right\rangle\text{d}t'+\left[1-f\max\left(L-\left|u\right|,0\right)\right]r_{0}^{2}$.

(S8)

The approximation in (S8) becomes more accurate for larger values of $T$ (same argument as with firing rates). The second term in the rhs arises from the fact that, for a single pattern presentation, the time interval during which both $t'$ and $t'+u$ fall in the presentation has duration $L-\left|u\right|$, when $-L<u<L$; otherwise, this never occurs. The first term in the rhs of Equation (S8) is sensitive to the spike timing within the pattern, which gives for every pair of spikes in each spike train (at times $t_{i}^{m}$ and $t_{j}^{m'}$, respectively) the following contribution:

$p^{2}\delta\left(u-t_{j}^{m'}+t_{i}^{m}\right)$,

(S9)

since each spike is presented with probability $p$. The term corresponding to the spikes replacing the missing ones is zero, because these spike times are randomly chosen and therefore probabilistically independent. Summing over all pairs of spikes and pattern presentations, we obtain

$\frac{1}{T}\int_{t-T}^{t} \mathbf{1}_{t'}\mathbf{1}_{t'+u}\left\langle S_{i}\left(t'\right)S_{j}\left(t'+u\right)\right\rangle\text{d}t'\approx fp^{2}\sum_{m,m'} \delta\left(u-t_{j}^{m'}+t_{i}^{m}\right)$.

(S10)

Altogether, this leads to

$C_{ij}\left(t,u\right)\approx fp^{2}\sum_{m,m'} \delta\left(u-t_{j}^{m'}+t_{i}^{m}\right)-fr_{0}^{2}\max\left(L-\left|u\right|,0\right)$.

(S11)

The second term in the rhs of Equation (S11) describes the “silence” during the pattern and applies to all coefficients $C_{ij}$ (even for $i=j$). Consequently, its contribution to the weight competition between pattern afferents is small. Moreover, this term corresponds to a much more spread function than delta functions in Equation (S11). Since spike-time correlations are used in convolutions to determine the matrix $\mathbf{A}$ in Equation (11) in the main text, sharper peaks in the correlogram give the leading order (when comparing functions with similar integral values). Ignoring that term, we obtain Equation (13) in the main text.

In a similar manner to the firing rates, the spike-time correlations here do not practically depend on time $t$ (as will be shown for the other models). In other words, the corresponding stochastic point process for the spike generation (including all inputs) can be considered to be second-order stationary with a good approximation.

### S1.3 Spike-time covariance structure for pattern model SJ

Jittering the spikes means that the ensemble average $\left\langle S_{i}\left(t'\right)S_{j}\left(t'+u\right)\right\rangle$ used to evaluate the cross-correlation will give, instead of a delta function as before for coincident spiking, the convolution of the respective jitter distributions that determine the time lag between spikes from each train. In the case of Gaussian jitters used for model SJ, Equation (S11) becomes (after neglecting the second “silence” term in the rhs)

$\begin{matrix} \frac{1}{T}\int_{t-T}^{t} \mathbf{1}_{t'}\mathbf{1}_{t'+u}\left\langle S_{i}\left(t'\right)S_{j}\left(t'+u\right)\right\rangle\text{d}t'\approx fp^{2}\sum_{m,m'} \int_{0}^{L} G\left(t'-t_{i}^{m},\sigma_{i}^{m}\right)G\left(t'+u-t_{j}^{m'},\sigma_{j}^{m'}\right)\text{d}t' \\ \approx fp^{2}\sum_{m,m'} G\left(u-t_{j}^{m'}+t_{i}^{m},\sqrt{\sigma_{j}^{m'}+\sigma_{i}^{m}}\right) \end{matrix}$,

(S12)

where the normalized Gaussian kernel function$G$ is given in Equation (15) in the main text. We allow the situation where the jittered spike times fall outside the borders of pattern presentations (in simulation too); the integration over $\left[0,L\right]$ above is somehow extended to its neighborhood. This leads to Equation Error: Reference source not found(14) in the main text. For Models SJ, the homogeneous contribution related to “silence” (second term with $\max\left(L-\left|u\right|,0\right)$ in the rhs of Equation (S11)) can be ignored when it is a more spread function compared to Gaussian jitters. In this case, the contribution to matrix $\mathbf{A}$ is much weaker than the rhs of Equation (S12) (provided $p$ is not negligible here). Note that an extra term due to the autocorrelation also applies for $i=j$; such terms are neglected when assuming a large number of inputs [Error: Reference source not found].

### S1.4 Spike-time covariance structure for pattern model R

Now we show how these results extend to a rate-modulated pattern. The fast covarying rate modulations exhibited by the intensities $\left\langle S_{i}\left(t\right)\right\rangle$ play the same role as the spike timing in model S; likewise, the integration over time performs the averaging as the ensemble average denoted by the angular brackets did. Here we denote by $\vartheta_{i}^{m}$ the whole collection of latencies for input $i$ (irrespective of pattern presentation), and by $\varsigma_{i}^{m}$ the respective spread widths. The joint probability of spikes, equivalent to Equation (S12) for model SJ, is given by the product of all Gaussian peaks:

$\left\langle S_{i}\left(t'\right)S_{j}\left(t'+u\right)\right\rangle=\sum_{m,n} a_{i}^{m}a_{j}^{n}\left\langle G\left(t'-\vartheta_{i}^{m},\varsigma_{i}^{m}\right)G\left(t'+u-\vartheta_{j}^{n},\varsigma_{j}^{n}\right)\right\rangle$,

(S13)

where the angular brackets relate to the randomness in generating the latencies $\vartheta_{i}^{m}$ and $\vartheta_{j}^{n}$ (recall that $p=1$ here). When integrating over time, we can swap the averaging and integration steps

.

(S14)

The approximation here relates again to negligible border effects at $t$ and $t-T$, which are neglected for sufficiently large $T$. For two independent input spike trains, the respective distribution of times $\vartheta_{i}^{m}$ and $\vartheta_{j}^{n}$ actually spans the whole Gaussian function, which gives its integral. Since it is normalized, we obtain

$\frac{1}{T}\int_{t-T}^{t} \left\langle S_{i}\left(t©\right)S_{j}\left(t©+u\right)\right\rangle\text{d}t©\approx\frac{1}{T}\sum_{m,n} a_{i}^{m}a_{j}^{n}$.

(S15)

The same dichotomy as in Equation (S3) between pattern presentation and background mode can be made. Within the pattern presentation, the contributions to Equation (S14) are determined by the relative latency differences and the shapes of the rate peaks. The result has a similar expression to Equation (S8) with $p=1$ and replacing the Dirac delta function by the Gaussian kernel in Equation (15)Error: Reference source not found:

.

(S16)

The “silence” term (cf. second term in the rhs of Equation (S11)) is ignored as it applies to all pattern inputs; however, for large spread widths ($\sigma_{i}^{m}>L/\sqrt{2}$), this term may not be negligible in the convolution, in contrast to model S. We then obtain Equation (17) in the main text.

### S1.5 Spike-time covariance structure for pattern models SB

Finally, we examine spike pattern activity where inputs are divided into two groups numbering $N_{1}$ and $N_{2}$, with the respective Gaussian distributions for the latencies with variance $\bar{\sigma}_{x}$ around the mean $\bar{t}_{x}$, where $x=1,\text{ }2$; in addition, we require $p=1$. We evaluate the mean cross-correlogram in the sense of population average between two inputs $i$ and $j$ taken randomly in groups $x$ and $y$, respectively. In the same manner as previously, we now evaluate the distribution of latencies $t_{i}$ and $t_{j}$ in each group:

,

(S17)

which is the expectation of the time lags (delta functions) averaged over each group. As before, the approximation relates to the integration boundaries. Performing the integrals, we get

,

(S18)

which is Equation (16) in the main text.

### S1.6 Model R + bursts for Fano factor >1

Our theoretical framework presented in the main text shows the independence of learning with respect to the Fano factor for $FF\leq1$. To complement the present study, we now consider patterns for which$FF>1$. To generate such input spike trains, we start from an initial Poisson spike train of Model R. For each spike thus generated, a burst of $k$ supplementary spikes is added with probability $q$. For a given time window, denoting by $n$ the original number of spikes of the Poisson spike train and by $b$ ($0\leq b\leq n$) the number of generated bursts, the number of spikes of the bursty train is $m=n+kb$. A Poisson spike train satisfies $\mathrm{E}\left[n\right]=\mathrm{var}\left[n\right]$ (i.e. $FF=1$) and, for a given $n$, as $b$ is binomially distributed, $\mathrm{E}\left[b|n\right]=qn$ and $\mathrm{var}\left[b|n\right]=q(1-q)n$. Therefore, one can easily demonstrate that $\mathrm{E}\left[m\right]=\mathrm{E}\left[n\right]\left(1+kq\right)$ and $\mathrm{var}\left[m\right]=\mathrm{E}\left[n\right]\left(1+k(k+2)q\right)$, leading to

$FF(k,q)=\frac{\mathrm{var}\left[m\right]}{\mathrm{E}\left[m\right]}=1+\frac{k(k+1)q}{1+kq}$.

(S19)

Clearly, we have $FF(k,q)\geq1$, and even $FF(k,q)>1$ when $k\geq1$ and $q>0$. In fact, this reasoning is also valid for $k=-1$, that is, when spikes are eliminated, and it can be seen that a spike train obtained by randomly eliminating spikes from a Poisson spike train is still a Poisson spike train ($FF(-1,q)=1$), which is not the case when spikes are added.

For the simulation shown in Fig. 2 and 3 (with a Poisson neuron), we have chosen $k=1$ and $q=1/4$, leading to $FF(1,1/4)=1.4$. To obtain the same mean firing rate as that of the baseline simulation, we use an initial Poisson spike train with rate $r(t)/\left(1+kq\right)=0.8r(t)$ to generate the bursty trains, and add the supplementary spikes are placed just 1 ms after the original ones to minimize the blurring of the time-dependent rate. The bursts hardly modify the cross-correlogram (see Fig. S2), therefore STDP leads to a similar weight structure than that of model R (see Fig. 3R1,2 in the main text). Recognition after learning is only slightly impaired (Mutual information = 0.12 bits versus 0.13 for model R, see Fig. 6 in the main text).

# S2. Complements on analysis of synaptic weight dynamics

## S2.1 Conditions for a stable equilibrium of the mean weight

Here we briefly recapitulate previous results [2, Error: Reference source not found] to study the homeostatic stability of the learning dynamics, namely the situation where the mean weight stabilizes; cf. Equation (18) to (20) in the main text. Averaging Equation (5) while ignoring the correlation terms $F_{i}$, we obtain the following dynamical equation that describes the evolution of the mean weight $J_{\text{av}}=\sum_{i} J_{i}/N$:

$\frac{dJ_{av}}{dt}\approx w^{i\text{n}}\nu_{\text{av}}+w^{\text{out}}\nu_{\text{out}}+\tilde{W}\nu_{\text{out}}\nu_{\text{av}}$,

(S20)

where the dependence on time is omitted. Using Equation Error: Reference source not found(4) in the main text that describes the spiking mechanism of the Poisson neuron, the output firing rate $\nu_{\text{out}}$ is approximated by

$\nu_{\text{out}}\approx\nu_{0}+NJ_{\text{av}}\nu_{\text{av}}$.

(S21)

Equation (S20) can thus be rewritten as a linear differential equation:

.

(S22)

Since the mean input firing rate is positive ($\nu_{\text{av}}>0$), this leads to the following necessary condition for a stable fixed point for the mean weight $J_{\text{av}}$

$$w^{\text{out}}+\tilde{W}\nu_{\text{av}}<0$$

(S23)

with equilibrium value

.

(S24)

The condition$w^{i\text{n}}>0$ ensures a positive equilibrium value in Equation (S24). When furthermore requiring stability irrespective of the input firing rate $\nu_{\text{av}}$, we obtain the conditions in Equation Error: Reference source not found(18) in the main text. Equations (19) and (20) are straightforwardly derived from Equations (S21) and (S24).

### S2.2 Competition between two input populations for a pattern of models SB and RB

Here we describe the relationship between the weight dynamics and the qualitative properties of matrix $\mathbf{A}$ in the case of patterns that consist of two populations of inputs, namely models SB (spike pattern) and RB (rate-modulated pattern). The temporal distribution of the pattern spikes for each group $x=1,\text{ }2$ is determined by the mean latency $\bar{t}_{x}$ and variance $\bar{\sigma}_{x}$, cf. Results. To evaluate the competition between the two input groups, we examine the evolution of the mean weights over each group, namely $N_{1}\bar{J}_{1}$ and $N_{2}\bar{J}_{2}$. This analysis is valid for both models SB and RB, since their population-averaged correlograms are theoretically identical. First, performing a population average (denoted by the overline) for the matrix $\mathbf{A}$ for model SB, we obtain the following matrix after combining Equations (11) and (16) in the main text, to obtain an equivalent equation to Equation (30):

,

(S25)

where for the sake of simplicity. Figure 4 gives a graphical interpretation of the convolutions that determine the matrix elements in Equation (S25), which illustrates the interplay between the time lag between the two groups and their temporal resolutions. Second, for model RB, all matrix elements of $\mathbf{A}$ are actually identical within each of the four quarters and correspond to Equation (17) with all amplitudes equal to $a_{i}^{m}=1$, which means that the expression for the population-averaged matrix $\bar{\mathbf{A}}$ in (S25) is also valid for model RB. The following analysis thus applies to both models SB and RB. We further define the following matrix where the elements of $\bar{\mathbf{A}}$ are multiplied by the number of elements of the group corresponding to the column index:

,

(S26)

such that an equivalent equation to Equation (21) in the main text can be rewritten to evaluate the relative evolution of $N_{1}\bar{J}_{1}$ and $N_{2}\bar{J}_{2}$:

.

(S27)

This equation, together with the homeostatic equilibrium that is considered to remain satisfied at all times:

,

(S28)

allows us to predict which input group wins the competition via a simplified fixed-point analysis for . First, we study the case when the corresponding fixed point is unstable: . The difference diverges in the opposite direction to the unstable fixed point, whose sign is the same as . Consequently, when the following condition is satisfied:

,

(S29)

the first group wins the competition and the second group is repressed (with initially homogeneous weights). Otherwise, the converse situation occurs: the second group takes over. Second, a similar analysis in the case of a stable fixed point shows that the condition in Equation (S29) also leads to the potentiation of the first group. We now study for models SB and RB when Equation (S29) is satisfied, to predict the outcome of the competition between the two input groups.

When both groups have comparable numbers of inputs and spread widths ($N_{2}\approx N_{1}$ and ) and, in addition, the first group arrives significantly before the second group (say ms), we have $\breve{\mathbf{A}}\sim\left(\begin{matrix} + & - \\ + & + \end{matrix}\right)$. This stems from the typical shapes of the curves of $\kappa\left(u,\bullet\right)$ in Fig. 2 (main text): $\breve{A}_{11}>0$, $\breve{A}_{22}>0$ and $\breve{A}_{21}>0$ while $\breve{A}_{12}<0$. In this form, the antidiagonal terms of opposite signs relate to the difference between the latencies and the asymmetry for the STDP learning window $W$. In general, Equation (S29) is then satisfied and STDP tends to select the first group in this case, as illustrated in Fig. 4SB3,6 and 4RB3,6 in the main text.

Now we consider that the two input groups have similar size ($N_{1}\approx N_{2}$), but distinct spread width: a sharper Gaussian for the second group (). This causes the matrix coefficients and to fade to zero, as illustrated by the less peaked curves in Fig. 2B in the main text, which results in $\breve{\mathbf{A}}\sim\left(\begin{matrix} 0 & 0 \\ 0 & + \end{matrix}\right)$. In this situation, Equation (S29) is not satisfied and STDP selects the second group instead of the earlier first group, see Fig. 4SB5,8 and 4RB5,8 in the main text. This result extends previous results about the effect of the spread width for two input groups that have no correlation between them [1].

Last, when $N_{2}>>N_{1}$ and , we have the following tendency: $\breve{\mathbf{A}}\sim\left(\begin{matrix} 0 & - \\ 0 & + \end{matrix}\right)$. The condition $\kappa\left(0,\bullet\right)>0$ means that narrowly correlated inputs are potentiated, which is always true for Hebbian STDP [2]. The positive values for favor within-group correlations (diagonal elements of $\breve{\mathbf{A}}$) and the mean weight of the second group is potentiated. Despite arriving earlier and having the same characteristics for their respective the Gaussian distributions (spread), the first group is not selected, as illustrated in Fig. 4SB4,7 and 4RB4,7 (main text) to be compared with Fig. 4SB3,6 and 4RB3,6 (main text). Note that, for a larger spread width $\bar{\sigma}_{2}>0$, becomes smaller and a larger misbalance $N_{2}>>N_{1}$ is necessary for the second group to be selected (data not shown). This is also true for a faster synaptic response kernel $\varepsilon$ that leads to more negative values of $\kappa\left(u,0\right)$ for small $u>0$ (black thin dashed-dotted line in Fig. 4) and thus weaker value for $\kappa\left(0,\sigma\right)$.

### S2.3 Theoretical evaluation of mutual information

For the calculation, we discretized the [2000,2500] s period into 25 ms time bins. Each of those bins could either correspond to the first 25ms of the pattern (or stimulus), case referred to as $s$, or not ($\bar{s}$), and could contain at least 2 postsynaptic spikes ($r$) or not ($\bar{r}$). The mutual information defined in Equation (25) the main text is:

.

(S30)

Note that in signal detection terms, the first term corresponds to “hits”, the second to “misses”, the third to “false alarms”, and the last one to “correct rejections”. A perfect detector would lead to ,$P(r,s)=P(s)=P(r)$ and . Therefore an upper bound on the mutual information is:

,

(S31)

which is the entropy of the stimulus. Here $P(s)=f\times25ms=0.0375$ leading to $MI_{\max}\approx0.23$ bits. In order to get analytical insight about the detection performance of the neuron, we evaluate the mutual information after convergence of learning and relate it to the learning parameters. We examine the idealized situation where all weights are either saturated or quiescent, namely the ratio of potentiated weights is $\theta\approx J_{\text{av}}^{*}/J_{\max}$. Outside pattern presentation, the rate is considered to be stable at $\nu_{\text{out}}\approx\nu_{\text{out}}^{\text{*}}$. During the pattern presentation, potentiated (pattern) inputs fire one spike on average, as used in our numerical simulations. We further consider that all potentiated inputs correspond to early spikes and that, consequently, the increase of the soma potential due to the pattern presentation entirely fits within the first 25 ms (i.e., $L/2$) of the presentation. This implies that the increase of input stimulation during these 25 ms corresponds to a mean rate of $\frac{2}{L}\approx2r_{0}$ Hz. It follows that the neuronal firing rate averaged over these 25 ms is ,

(S32)

after using Equations (19) and (20) in the main text. Increasing the value of $\nu_{\text{pattern}}$ implies a higher probability to fire spikes right after the start of the pattern presentation.

More precisely, we can approximate the probability for the Poisson neuron to fire at least 2 spikes during the first 25 ms (i.e., event $r$) of the pattern presentation by:

.

(S33)

The function $1-\left(1+x\right)\exp\left(-x\right)$ increases with $x\geq0$. It follows that should be maximized for a better detection.

On the other hand, should be small to minimize false alarms, as explained in the main text. This explains why the combination of a small value for $w^{i\text{n}}>0$ together with inhibition via $\nu_{0}<0$ gives the best results, while keeping $w^{\text{out}}+\tilde{W}r_{0}$ unchanged.

### S2.4 Link to previous results for $w^{i\text{n}}=w^{\text{out}}=0$

It is worth mentioning that, although they greatly increase robustness, the homeostatic terms are not necessary. This particular case has been studied by [3] using numerical simulation for a LIF neuron and additive STDP. In agreement with prediction, a slightly negative value for $\tilde{W}<0$ leads to a stable equilibrium value of the output firing rate $\nu_{\text{out}}^{\text{*}}\approx0$ in Equation (20) in the main text, which implies that the neuron is almost always silent and corresponds to all weights depressed to zero. However, this prediction on the output firing rate ignores spike-time correlations, i.e., is strictly valid only when the neuron is fed with inputs with a lot of variability in their spike times, i.e., outside patterns. Parameters can be chosen such that some weights become potentiated, provided input correlations are sufficiently strong; in this case, the covariance term $F^{W}$ in the learning equation may become the leading order when getting close to the homeostatic equilibrium ($\nu_{\text{out}}^{\text{*}}\approx0$). It follows that it is possible to obtain a bimodal distribution of weights, synonymous with a fine-tuned selectivity such that the neuron fires if (and only if) the pattern is presented. This configuration required to adjust the parameters so that the orders of the third and fourth terms in the learning equation can be comparable; otherwise, all input weights become depressed, as was reported in that previous study.

A positive consequence of the homeostatic mechanism introduced here is that learning can be successful with spreads of Gaussian peaks over 20 ms, whereas jitters of the order of 5 ms completely compromised learning in that study.

# S3 Numerical simulation

All the simulations were run with Brian (http://www.briansimulator.org/), a Python-based clock-driven spiking neural network simulator [4]. The input spike trains were prepared before the simulations using MATLAB R2007b (Mathworks, Natick, MA). All the code has been made available on ModelDB (<http://senselab.med.yale.edu/modeldb/>).

### S3.1 Leaky Integrate-and-Fire (LIF) neuron model

In some numerical simulations, we have used a classical LIF neuron, whose soma (membrane) potential $V$ is governed by the following ordinary differential equation:

$\tau_{m}\frac{dV}{dt}=-V+E_{l}+RI\left(t\right)$,

(S34)

where $E_{l}$ is its resting value, $\tau_{m}$ is the membrane time constant, $R$ is its resistance and $I\left(t\right)$ is the input current caused by incoming spikes:

,

(S35)

where $\hat{t}_{i}^{m}$ denote the spike times arriving at synapse $i$, $\tau_{s}=2.5$ ms is the synaptic time constant (chosen to comply with the parameters for the Poisson neuron model). Whenever the threshold $V_{t}=-54$ mV is reached, a spike is emitted and the soma potential is reset to $V_{r}=-60$ mV and clamped there for a refractory period of 1 ms. The differential equation is solved numerically (Euler method) with a time step of 0.1 ms. The synaptic and membrane time constants were chosen to match those of the Poisson neuron model, as well as the resistance such that a similar number of weights are potentiated at the end of the learning epoch.

### S3.2 Simulation Parameters

For convenience we gather here all the numerical parameters used in the simulations (unless said otherwise):

| Input spike trains | | |
| --- | --- | --- |
| $N$ | 1,000 | Number of afferents. |
| $N_{0}$ | 500 | Number of afferents involved in the pattern. |
| $r_{0}$ | 20 Hz | Mean firing rate of all pattern and non-pattern afferent inputs. |
| $f$ | 1.5 Hz | Pattern presentation frequency, avoiding two successive pattern presentations. This was done by discretizing time with time bins of length L. Each one had a probability of 0 of containing the pattern if the preceding time bin did, and of $Lf/(1-Lf)$ otherwise. |
| $L$ | 50 ms | Pattern duration. |
| $p$ | 1 | Probability of presence for each spike in model S and each Gaussian in model R. |
| STDP | | |
| $\eta$ | 10^-3^ | Learning rate. $\eta<<1$ for slow learning speed. |
| $\tau^{+}$ | 17 ms | Time constant for the exponential LTP part of STDP. |
| $\tau^{-}$ | 34 ms | Time constant for the exponential LTD part of STDP. |
| $a^{+}$ | 1.0x$J_{\text{max}}$ | Magnitude of the exponential LTP part of STDP. |
| $a^{-}$ | 0.82x$J_{\text{max}}$ | Magnitude of the exponential LTD part of STDP. |
| $\gamma$ | 0 | This parameter interpolates between additive ($\gamma=0$) and multiplicative ($\gamma=1$) STDP [5]. |
| $w^{\text{out}}$ | -1.0x$J_{\text{max}}$ | Homeostatic LTD term. |
| $w^{\text{in}}$ | 0.5x$J_{\text{max}}$ | Homeostatic LTP term. |
| Poisson neuron model | | |
| $\varepsilon\left(t\right)$ | $\begin{matrix} \begin{matrix} 1/(\tau_{d}-\tau_{r})\cdot\\ \left(\exp\left(-t/\tau_{d}\right)-\exp\left(-t/\tau_{r}\right)\right) \end{matrix} \\ \begin{matrix} with & \tau_{d}=10ms, & \tau_{r}=2.5ms \end{matrix} \end{matrix}$ | EPSP kernel. |
| $\nu_{0}$ | -200 Hz | Spontaneous firing rate (here inhibition). |
| $J_{\text{max}}$ | 0.1125 | Maximal synaptic weight. |
| $J_{\text{initial}}$ | Uniformly distributed in  [0, 0.035] | Initial synaptic weights. |
| LIF neuron model | | |
| $\tau_{m}$ | 10 ms | Membrane time constant. |
| $\tau_{s}$ | 2.5 ms | Synaptic time constant. |
| $E_{l}$ | -70 mV | Resting potential. |
| $V_{t}$ | -54 mV | Threshold. |
| $V_{r}$ | -60 mV | Reset potential. |
| $RJ_{\text{max}}$ | 2.8 mV | Maximal synaptic weight. |
| $RJ_{\text{initial}}$ | Uniformly distributed in  [0, 0.7] mV | Initial synaptic weights. |

# Supplemental figures

Figure S1. Typical evolution of the plastic weights subject to additive STDP with model R (Fig. 3R1,2). The grey traces represent simulated individual weights (using Poisson neurons in discrete time), the black solid line being their mean $J_{\text{av}}$. The black dashed line indicates the predicted equilibrium value $J_{\text{av}}^{\text{*}}$. An upper bound equal to 0.1125 was enforced onto the weights.

Figure S2. (Similar to Fig.1R2) Crosscorrelogram between spike times of afferents 1 and 2 for a Model R with bursts (Fano factor $FF=1.4$), see Section S1.2. Again the circles represent the simulation, and the dotted and dashed lines the theoretical predictions (the dashed lines involve an additional approximation compared to the dotted line that is more accurate (compare Equations (41) and (13), respectively)). The cross correlogram is hardly affected by the bursts.

Figure S3. Emerged input selectivity after learning ($t\approx2000$ s) for a Poisson neuron trained with model R with bursts ($FF=1.4$). Plots are similar to those of Fig. 3. STDP leads to a similar weight structure (inset) to that of model R, and the resulting selectivity is hardly impaired (bottom).

Figure S4. Graphical illustration of the convolution of $\kappa(u,0)$ in Equation (30) with spike-time correlograms to obtain matrix $A$ (cf. Equation (S25)) for models SB and RB. The Gaussian functions (grey curves) represent the idealized population-averaged cross-correlograms $\bar{C}_{xy}(u)$ between the groups of pattern inputs: the solid (narrowly correlated group, $\bar{\sigma}=2$ ms) and dotted (more broadly correlated group, $\bar{\sigma}=5$ ms) lines corresponds to the same group $x=y$; the dashed-dotted line to the early group $x$ and the late group $y$; and the dashed line to the converse situation. The variance for each Gaussian-like cross-correlogram is determined by the temporal resolutions of the corresponding groups (via $\bar{\sigma}_{x}$), cf. Equation (S25). As a result, not only the relative timing of the two groups, but also their spreads matter to determine the weight competition.

# References

1. [1] Kistler WM, van Hemmen JL (2000) Modeling synaptic plasticity in conjunction with the timing of pre- and postsynaptic action potentials. Neural Comput 12: 385–405.
2. [2] Kempter R, Gerstner W, van Hemmen JL (1999) Hebbian learning and spiking neurons. Phys Rev E 59: 4498-4514.
3. [3] Masquelier T, Guyonneau R, Thorpe SJ (2008) Spike timing dependent plasticity finds the start of repeating patterns in continuous spike trains. PLoS ONE 3: e1377.
4. [4] Goodman D, Brette R (2008) Brian: a simulator for spiking neural networks in python. Front Neuroinformatics 2: 5.
5. [5] Gütig R, Aharonov R, Rotter S, Sompolinsky H (2003) Learning input correlations through nonlinear temporally asymmetric hebbian plasticity. J Neurosci 23: 3697–3714.
